# Biological Evaluation of Platinum(II) Sulfonamido Complexes: Synthesis, Characterization, Cytotoxicity, and Biological Imaging

**DOI:** 10.1155/2022/7821284

**Published:** 2022-09-13

**Authors:** Charini Maladeniya, Taniya Darshani, Sameera R. Samarakoon, Frank R. Fronczek, W. M. C. Sameera, Inoka C. Perera, Theshini Perera

**Affiliations:** ^1^Department of Chemistry, University of Sri Jayewardenepura, Nugegoda, Sri Lanka; ^2^Institute of Biochemistry, Molecular Biology and Biotechnology, University of Colombo, Colombo, Sri Lanka; ^3^Department of Chemistry, Louisiana State University, Baton Rouge 70803, Louisiana, USA; ^4^Institute of Low Temperature Science, Hokkaido University, N19-W8 Kita-ku, Sapporo, 060-0819, Hokkaido, Japan; ^5^Department of Zoology and Environment Sciences, University of Colombo, Colombo, Sri Lanka

## Abstract

Platinum-based compounds are actively used in clinical trials as anticancer agents. In this study, two novel platinum complexes, (*C*1 = [PtCl_2_(*N*(SO_2_quin)dpa)], *C*2 = [PtCl_2_(*N*(SO_2_azobenz)dpa)]) containing quinoline and azobenzene appended dipicolylamine sulfonamide ligands were synthesized in good yield. The singlet attributable to methylene CH_2_ protons of the ligands of C1 and C2 appears as two doublets in ^1^H NMR spectra, which confirms the presence of magnetically nonequivalent protons upon coordination to platinum. Structural data of *N*(SO_2_quin)dpa (L1), *N*(SO_2_azobenz)dpa (L2) and PtCl_2_(*N*(SO_2_quin)dpa) confirmed the formation of the desired compounds. Time-dependent density functional theory calculations suggested that the excitation of L1 show quin-unit-based *π⟶π*^*∗*^ excitations (i.e., ligand-centered charge transfer, LC), while C1 shows the metal-ligand-to-ligand charge-transfer (MLLCT) character. L1 displays intense fluorescence from the ^1^LC excited state, while C1 gives phosphorescence from the ^3^LC state. Mammalian cell toxicity of ligands and complexes was assessed with NCI–H292 nonsmall-cell lung cancer cells. Further, C1 and C2 showed significantly low IC_50_ values compared with *N*(SO_2_azobenz)dpa and PtCl_2_(*N*(SO_2_quin)dpa). Fluorescence imaging data of both ligands and complexes revealed the potential fluorescence activity of these compounds for biological imaging. All four compounds are promising novel candidates that can be further investigated on their usage as potential anticancer agents and cancer cell imaging agents.

## 1. Introduction

The field of medicinal chemistry has paid significant attention to the design of anticancer drugs in recent years. Since the serendipitous discovery of cisplatin [1] in the 1960s, the impact of metal complexes in the treatment of cancer has been tremendous. Apart from many advantages that metal-containing complexes offer over conventional carbon-based compounds, such as the ability to coordinate ligands in a three-dimensional configuration, the interesting electronic properties that transition metals impart, enable them to serve as probes in the design of anticancer agents [2].

Platinum(II) complexes have the advantages of inertness, low coordination number and specific binding of limited centers in proteins and nucleic acids [3]. The inertness of platinum(II) complexes evades unnecessary covalent reactions with nucleotides [4]. Most platinum(II) complexes have a high affinity toward N^7^ of purines, specifically guanine in nucleic acids [5]. The underlying key factor of the antitumor effect of platinum-based compounds is ligand exchange kinetics. Due to slow ligand exchange behavior, the kinetic stability of the relevant platinum compounds is high; ligands oriented in the *trans* position are rapidly converted than those in the *cis* position of platinum(II) compounds [6] in complexes where *cis* and *trans* are used to designate the relative position of two identical ligands/donor atoms. Due to these important insights, platinum(II) plays a major role in the success of the antitumor activity and may perhaps still be the best single type of anticancer drug which is active against a wide range of cancers.

On the other hand, transition metal complexes have also attracted attention as intracellular sensors and bioimaging agents due to specific characteristics, such as high photostability, long-lived phosphorescence and large Stokes shifts associated with transition metals [7]. Furthermore, transition metal complexes containing *π*-conjugated ligands exhibit interesting two-photon absorption behavior [7]. Fluorescence probes can be widely used as visualizing agents of an individual cell or sub cellular components such as DNA [8]. When a specific fluorophore is chemically or noncovalently bound to a live cell or a sub-component of a cell, images of the behavior of the cell or the cell component may be utilized to detect the behavior of cancer cells [9].

In this study, we attempted to utilize the application of transition metal complexes both as fluorescence probes and as anticancer agents. Metal-to-ligand charge-transfer (MLCT) excitation of the transition metal complexes facilitates higher delocalization of excited electrons throughout the ligand [10]. In the presence of a heavy transition metal, Pt, for instance, spin-orbit coupling allows singlet-triplet intersystem crossing. Thus, the emission occurs from the lowest excited triplet state. Sulfonamides were chosen because sulfonamide groups are considered as a pharmacophore and are present in many biologically active molecules [11]–[15], while their most important property related to modern aspects of medicine is the ability to act as anticancer agents [16].

**Figure 1:**
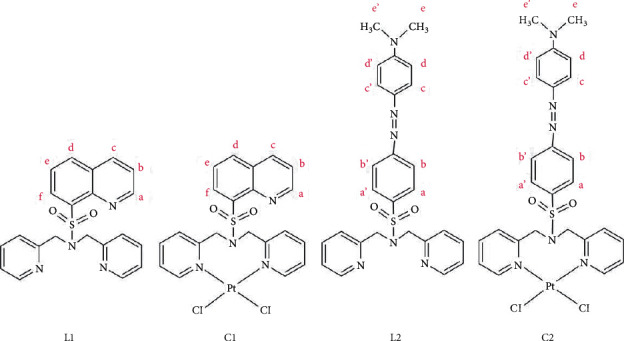
The line diagram of proposed ligands ((NSO_2_quin)dpa (L1), (N(SO_2_azobenz)dpa (L2)) and platinum complexes (PtCl2((NSO_2_quin)dpa) (C1), PtCl2((NSO_2_azobenz)dpa) (C2)).

In designing our metal complexes, we took into consideration the lipophilic nature of dipicolylamine ligands in facilitating the uptake of metal complexes by cell membranes [17] as well as of potential characteristics that the *R* group (Figure 1) may impart. We note that quinoline is a pharmacologically valuable compound present in biologically active natural and synthetic compounds [18] and that interesting bioactivities, such as antibacterial, antifungal, anti-inflammatory, antimalarial as well as anticancer activities have been reported [19, 20]. The broader anticancer activity of quinoline derivatives has been used for various types of cancers, including breast, prostate, gastrointestinal tract, colon and liver [21] while quinoline-based camptothecin and its analogues have been clinically used as anticancer drugs [22].

Azobenzene shows strong electronic absorption and has thereby been used as a chromophore [23]. The most interesting property of azobenzene as a chromophore is reversible isomerization between *cis* and *trans* [23]. The important phenomena displayed by azo aromatic compounds are the incorporation of these chromophores into various molecular systems, such as polyelectrolytes, inorganics, surfaces, and biopolymers such as DNA [23]. Due to the rigid and *π* electrons delocalization of the aromatic ring structure of azobenzene, it can be utilized as a fluorophore.

This study explores the possibility of using Pt(II) complexes in 8-membered chelate rings toward biological applications. Herein, we report the extensive spectral characterization as well as computational studies of the compounds (Figure 1) supported by preliminary biological studies, which may guide specific research related to the dipicolylamine scaffold in the near future.

## 2. Experimental Section

### 2.1. Starting Materials

K_2_(PtCl_4_), DMSO, quinoline-8-sulfonyl chloride, 4-(dimethylamino)azobenzene-4′-sulfonyl chloride, di(2-picolyl)amine, anhydrous sodium sulfate, dioxane, methanol, dichloromethane, acetone, chromasolv water were obtained from Sigma Aldrich, USA. NCI–H292 (human nonsmall-cell lung cancer cell line) and MRC-5 (human lung fibroblast cell line) were obtained from American Type Culture Collection. All chemical compounds and solvents were of analytical grade and were used without further purification.

### 2.2. Methodology

^1^H NMR spectra were recorded in DMSO-d_6_ on a Bruker 400 MHz spectrometer. Peak positions are relative to tetramethylsilane (TMS) as reference. All NMR data were processed with TopSpin 3.2 and MestReNova software. Single crystals were placed in a cooled nitrogen gas stream on a Bruker Kappa Apex-II DUO diffractometer equipped with Mo *Kα* radiation (*λ* = 0.71073 Å) (*N*(SO_2_quin)dpa and [PtCl_2_(*N*(SO_2_quin)dpa)]) or Cu *Kα* (*λ* = 1.54184 Å) radiation (*N*(SO_2_azobenz)dpa). Refinement was performed by full-matrix least-squares methods using SHELXL (Sheldrick (2008)), with H atoms in idealized positions. Molecular graphics are drawn using *ORTEP-3* for windows. Electronic spectra of the ligands and metal complexes were obtained on GENESIS 10S UV-Vis spectrophotometer. The spectral range was 190 nm–700 nm. Spectra were obtained in methanol with baseline correction. FTIR spectra were recorded on a Thermo Scientific Nicolet iS10 spectrophotometer. ATR spectra were obtained within the 4000–600 cm^−1^ spectral range. Spectral data were processed with OMNIC software. Emission spectra were obtained in methanol on a Thermo Scientific Lumina spectrophotometer. A 150 W Xenon lamp was used as the excitation source. Spectral data were processed with Luminous software.

### 2.3. Synthesis

In order to synthesize the new metal complexes, PtCl_2_(DMSO)_2_ precursor was prepared using K_2_[PtCl_4_] as the starting material according to a known procedure [24]. We followed the methods of Subasinghe et al. [25] with the sulfonyl chloride having the desired *R* groups in the synthesis of L1 and L2.

#### 2.3.1. Synthesis of N(SO_2_quin)dpa (L1)

A solution of quinoline-8-sulfonyl chloride (0.290 g, 1.25 mmol) in 6.25 ml of dioxane was added dropwise over a period of 2 h to a solution of N(H)dpa (0.513 g, 2.5 mmol) in 25 ml of dioxane at room temperature. The reaction mixture was stirred at room temperature for 24 h and filtered to remove any precipitate. Thereafter, the dioxane was completely removed by rotary evaporation. Slightly acidic water (30 ml, pH∼5) was then added to the resulting compound, and the product was extracted into CH_2_Cl_2_ (2 × 25 ml). The CH_2_Cl_2_ extracts were combined, washed with water (2 × 25 ml) and taken to dryness to give a green color precipitate (0.417 g, 85%). FTIR (cm^−1^): 930 (S–N). ^1^H NMR signals (ppm) in DMSO-d_6_ are 9.01 (d, 1H), 8.48 (d, 1H), 8.38 (d, 1H), 8.24 (d, 1H), 8.21 (d, 2H, H6/H6'), 7.70–7.63 (m, 2H), 7.51 (t, 2H, H4/H4′), 7.11 (d, 2H, H3/H3'), 7.07 (t, 2H, H5/H5′), 4.81 (s, 4H, CH_2_). Anal. Calc. for C_21_H_18_N_4_O_2_S·CH_3_CN : C,64.02; H, 4.91; N, 16.23. Found: C: 64.19%, H: 4.76%, N: 15.58%. Crystals suitable for single crystal X-ray diffraction were grown by slow evaporation of the compound in acetonitrile.

#### 2.3.2. Synthesis of PtCl_2_(N(SO_2_quin)dpa) (C1)

A solution of *N*(SO_2_quin)dpa (0.039 g, 0.1 mmol) in 10 ml of ethanol was added to a solution of PtCl_2_(DMSO)_2_ (0.042 g, 0.1 mmol) in 10 ml ethanol at room temperature. The reaction mixture was stirred at 50°C for 12–18 h and the resultant precipitate was collected on a filter paper and dried. The product was a green powder (0.035 g, 54%). FTIR (cm^−1^): 894 (S–N). ^1^H NMR signals (ppm) in DMSO-d_6_: 9.26 (d, 2H, H6/H6'), 9.25 (1H), 8.66–8.61 (m, 2H), 8.44 (d, 1H), 7.97 (t, 2H, H4/H4′), 7.88 (t, 1H), 7.82–7.78 (m, 1H), 7.70 (d, 2H, H3/H3'), 7.52 (t, 2H, H5/H5′), 5.95 (d, 2H, *exo*-H), 5.61 (d, 2H, *endo*-H). Anal. Calc. for C_21_H_18_Cl_2_N_4_O_2_PtS·H_2_O : C,37.4; H, 2.99; N, 8.31. Found: C: 37.59%, H: 3.23%, N: 8.48%. Crystals suitable for single-crystal X-ray diffraction were grown by mixing two solutions of the ligand and platinum precursor (12.5 mM each) in acetonitrile.

#### 2.3.3. Synthesis of N(SO_2_azobenz)dpa (L2)

*N*(SO_2_azobenz) dpa ligand has been previously synthesized by a one-step reaction using THF and Et_3_N [26]. However, we note that the yield of the compound is significantly high in the procedure proposed herein. A solution of 4-(dimethylamino)azobenzene-4′-sulfonyl chloride (0.830 g, 2.5 mmol) in 12.5 ml of dioxane was added drop wise over a period of 2 h to a solution of N(H)dpa (1.027 g, 5 mmol) in 50 ml of dioxane at room temperature. The reaction mixture was stirred at room temperature for 24 h and then filtered to remove any precipitate. Thereafter, the dioxane was completely removed by rotary evaporation. Slightly acidic water (30 ml, pH∼5) was added to the resulting compound, and the product was extracted into CH_2_Cl_2_ (2 × 25 ml). The CH_2_Cl_2_ extracts were combined, washed with water (2 × 25 ml), and taken to dryness to give a red color precipitate (1.188 g, 98%). FTIR (cm^−1^): 917 (S–N). ^1^H NMR signals (ppm) in DMSO-d_6_ are 8.36 (d, 2H, H6/H6'), 7.92 (2H), 7.85–7.83 (m, 4H), 7.67 (t, 2H, H4/H4′)), 7.28 (d, 2H, H3/H3'), 7.19 (t, 2H, H5/H5′), 6.86 (d, 2H), 4.58 (s, 4H, CH_2_), 3.10 (s, 6H, CH_3_). Anal. Calc. for C_26_H_26_N_6_O_2_S : C,64.18; H, 5.39; N, 17.27. Found: C: 63.96%, H: 5.38%, N: 17.08%. Crystals suitable for single-crystal X-ray diffraction were grown by slow evaporation of the compound in methanol.

#### 2.3.4. Synthesis of PtCl_2_(N(SO_2_azobenz)dpa) (C2)

A solution of *N*(SO_2_azobenz)dpa (0.049 g, 0.1 mmol) in 10 ml of ethanol was added to a solution of PtCl_2_(DMSO)_2_ (0.042 g, 0.1 mmol) in 10 ml ethanol at room temperature. The reaction mixture was stirred at 50 ^0^C for 12–18 h. The resultant precipitate was collected on a filter paper and dried. The product was a dark red powder (0.046 g, 62%). FTIR (cm^−1^): 879 (S–N). ^1^H NMR signals (ppm) in DMSO-d_6_ are 9.26 (d, 2H, H6/H6'), 8.19 (d, 2H), 7.98 (2H), 7.97 (t, 2H, H4/H4′), 7.88 (d, 2H), 7.70 (d, 2H, H3/H3'), 7.52 (t, 2H, H5/H5′), 6.88 (d, 2H), 6.04 (d, 2H, *exo*-H), 5.28 (d, 2H, *endo-*H), 3.11 (s, 6H, CH_3_). Anal. Calc. for C_26_H_26_Cl_2_N_6_O_2_PtS·H_2_O : C,40.52; H, 3.66; N, 10.91. Found: C: 40.58%, H: 3.73%, N: 10.92%. Attempts to obtain crystals suitable for single-crystal X-ray diffraction were not successful.

### 2.4. Computational Methods

Ground state and excited state structures were fully optimized by using the Gaussian 16 program [27]. The PBE1PBE [28] functional, employing the Grimme's dispersion [29], and the Becke-Johnson damping was used for ground-state structure optimizations. The SDD [30, 31] basis set and associated effective core potentials were used for Pt. The det2-TZVP [32, 33] basis sets were applied for the remaining atoms. An implicit solvation model, specifically the polarizable continuum model (PCM) [34–36] was used with methanol (*ε* = 32.613) solvent. Vertical excitations and the lowest excited single and triplet states were calculated using the time-dependent density functional theory (TDDFT). For this purpose, the *ω*B97XD [37] functional was used. Also, the basis sets described above and the PCM were employed. The nonequilibrium PCM solvation and the equilibrium PCM solvation approaches were applied to compute the singlet vertical excitations and to optimize the excited states, respectively. For TDDFT calculations, the “UltraFine” integration grid and the two-electron integral accuracy parameter of 12 were used. Vibrational frequency calculations, at 298.15 K and 1 atm were performed to confirm that the optimized ground state or excited structures were local minima (i.e., no imaginary frequencies). Conformational analysis of L1 was performed using the GMMX method, employing the MMFF [38] force field. All located conformers were fully optimized using the PBE1PBE-D3BJ method, det2-TZVP basis sets, and the PCM in the Gaussian 16 program. The most stable conformer was used for calculating the excited states.

Drug likeness and target prediction: Predicting absorption, distribution, metabolism and excretion (ADME) is an important early step in the process of drug discovery. Structures of L1, L2, C1, and C2 were submitted to SwissADME web-based platform (https://www.swissadme.ch/) to predict the pharmacokinetics and the drug likeness of the synthesized ligands and their Pt complexes [39]. Furthermore, ligands and complexes were analyzed using SwissTargetPrediction tool (https://www.swisstargetprediction.ch/) to understand the probable macromolecular targets of them leading to their activity *in vitro* [40].

### 2.5. Biological Assays

#### 2.5.1. Cytotoxicity Assessment

The *in vitro* cytotoxic effect of the synthesized novel ligands and their platinum complexes was evaluated on nonsmall-cell lung cancer cell line (NCI–H292) and human lung fibroblast cell line MRC-5 (normal lung fibroblast cells as a control) by Sulforhodamine B (SRB) assay. NCI–H292 and MRC-5 cells (5 × 10^3^/well) were plated in 96-well cell culture plates with Dulbecco's Modified Eagle Medium (DMEM; Sigma Aldrich D5648) supplemented with 10% fetal bovine serum (FBS) and incubated for 24 h at 37°C under 95% air with 5% CO_2_. The medium was then removed and replaced with the fresh medium containing different concentrations of the compound (1.25, 2.5, 5, 10, and 20 *μ*g/ml) in triplicate. The treated plates were then incubated for 24, 48, and 72 h. After the incubation period, cells were fixed with trichloroacetic acid (TCA) solution and incubated at 4°C for 1 h. Then, the cells were washed five times with water and stained with SRB solution for 15 min at room temperature. After incubation, the dye was removed by rinsing the cells five times with 1% acetic acid and the plate was air-dried. Then unbuffered Tris-base was added to each well and the plate was placed on a shaker for 1 h at room temperature. The absorbance values were taken at 540 nm, and the result was expressed as percentage cell viability (mean of control group–mean of treated group/control group × 100%). IC_50_ values were calculated using the software GraphPad Prism 6.0.1.

#### 2.5.2. Fluorescence Activity

The stained *Allium cepa* cells were incubated in maximum tolerable concentration (1 mg/ml) of *N*(SO_2_quin)dpa, *N*(SO_2_azobenz)dpa), and PtCl_2_(*N*(SO_2_azobenz)dpa in DMSO solution for 10 minutes at room temperature and observed with the aid of an Olympus BX51 epifluorescence microscope. Fluorescent micrographs were obtained with an Olympus DP70 and analyzed using Olympus Stream software.

## 3. Results and Discussion

### 3.1. Structural Results

**Figure 2:**
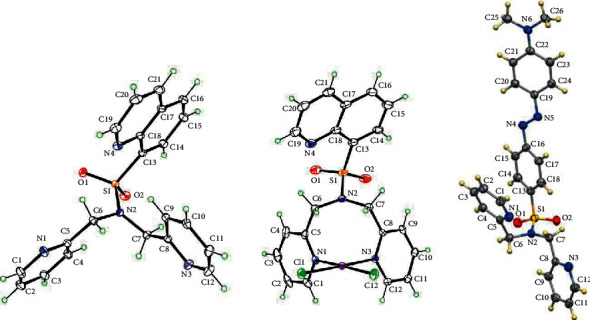
ORTEP of N(SO_2_quin)dpa (L1, left), PtCl2(N(SO_2_quin)dpa) (C1, middle), and N(SO_2_azobenz)dpa (L2, right). Thermal ellipsoids are drawn with 50% probability.

Crystal data and structure refinement for *N*(SO_2_quin)dpa, PtCl_2_(*N*(SO_2_quin)dpa) and *N*(SO_2_azobenz)dpa are provided in Table 1. Key structural parameters of the ground-state optimized structures and X-ray structures are summarized in Table 2. In general, computed structures are in agreement with the X-ray structures (Figure 2). The S1-N2 bond length in the un-coordinated ligands is ∼1.62 Å (Table 2) and is comparable with the reported values in related compounds in previous studies [41, 42]. Furthermore, the S1-N2 bond length in the ligand is not significantly different from that of the platinum complex as the ligand serves as a bidentate ligand, unlike in the case of Re(CO)_3_ complexes having similar ligands [42] Due to the conjugation of lone pair in sulfonamide nitrogen with S=O across the S–N bond, noncoordinated or weakly coordinated sulfonamide groups show significant double bond character [43].

The structural data revealed that newly formed S–N bond has no considerable impact on existing bonds. S–O bond lengths are similar to normal *sp*^2^ hybridized S=O bond length in SO_2_ (∼1.43 Ǻ) [44]. Furthermore, the bond length between methylene carbon and N2 (∼1.46 Ǻ) is similar to the normal *sp*^3^ hybridized C–N bond length. The normal bond length of sulfur and carbon in sulfonamide compounds (∼1.77 Å) is similar to S–C13 bond length. The individual aromatic C–C bond length of benzene ring in quinoline group is ∼1.4 Å. Thus, the two rings in quinoline group are coplanar. Depending on the hybridization, all the other bond lengths are in normal range.

### 3.2. ^1^H NMR Characterization

The assignment of the peaks related to the dipicolylamine moiety of the novel *N*(SO_2_quin)dpa ligand was done with the aid of data from previous studies. The most downfield signal for dipicolylamine (8.21 ppm) was observed for H6/6' proton, which is adjacent to the pyridyl nitrogen. Doublets of H3/3′ and triplets of H5/5' are close to each other and the position of signals for H3/3′ and H5/5' can interchange depending on the substituent group attached to the ring. A triplet for H4/4′ was observed at 7.51 ppm due to the para position to nitrogen in pyridyl ring (Table 3).

**Figure 3:**
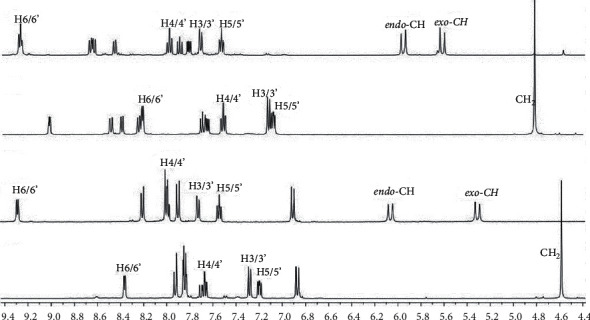
^1^H NMR spectra of *N*(SO_2_azobenz)dpa (a) and PtCl_2_ (*N*(SO_2_azobenz)dpa), (b) *N*(SO_2_quin)dpa, (c) and PtCl_2_ (*N*(SO_2_quin)dpa) (d) in DMSO-d_6_.

Protons in the quinoline ring were assigned using known spectra of related compounds. More deshielded doublets for Ha proton are located in 9.01 ppm position due to Ha proton close proximity to nitrogen atom (Figure 3). The proton which is located in para position of quinoline nitrogen atom (Hc) possesses the next highest deshielded signal (8.48 ppm). Doublets of Hc and Hd are close to each other. The signal for Hf appears at 8.24 ppm, which is close to sulfur atom in sulfonyl group. In the ligand, a singlet (4.81 ppm) is observed due to protons in the methylene groups because the two methylene groups are magnetically equivalent. Peaks appearing at 3.3, 2.5, 4.8, and 3.5 ppm are due to residual solvents of water, DMSO, dichloromethane and dioxane, respectively.

In ^1^H NMR spectra of *N*(SO_2_azobenz)dpa andPtCl_2_(*N*(SO_2_azobenz)dpa), doublets of Hc proton peaks appear in a more deshielded position in comparison with other protons (Figure 3). The signals for Hb and Ha protons were observed in the region of 7.85–7.83 ppm in the ^1^H NMR spectrum of *N*(SO_2_azobenz)dpa. In general, relatively a more upfield doublet was observed for Hd protons due to the lower effect of dimethyl groups.

### 3.3. FTIR Analysis

In the IR spectrum of L1, the short absorption band at 3059 cm^−1^ could be due to the asymmetric stretching vibration of the C–H bond in aromatic rings [45]. A narrow and short absorption band at 2856 cm^−1^ can be assigned to the C–H asymmetric stretching vibration of aliphatic systems. Strong absorption bands peaking at 1140 cm^−1^ and 1328 cm^−1^ correspond to the symmetric stretching vibrations and asymmetric vibrations of the O=S=O group [46], respectively. A collection of absorption peaks between 1211 cm^−1^ to 1590 cm^−1^ are due to the symmetric and asymmetric stretching vibrations of C=C bonds in aromatic rings and C=N stretching vibrations in the ligand. Stretching vibration due to the S–N bond can be obtained as a strong absorption peak at 930 cm^−1^ [47]. Most of the peaks in the spectrum of PtCl_2_(*N*(SO_2_quin)dpa) complex have slightly shifted in comparison with that of the ligand peaks. The peak due to the S–N stretching vibration of the ligand has slightly shifted to a lower frequency in the complex 894 cm^−1^ (Table S1, Supporting Information).

In the IR spectrum of L2, the absorption peak at 917 cm^−1^ was attributed to the stretching vibration of the S–N bond. The characteristic symmetric and asymmetric stretching vibrations of the O=S=O group in L2 can be observed at 1141 and 1330 cm^−1^, respectively. The S–N absorption peak of PtCl_2_(*N*(SO_2_azobenz)dpa) has shifted toward a lower frequency in comparison with that of the ligand spectrum. In a previous study where Pt is bound to dpa-sulfonamide moiety, we observed a slight shift toward lower frequency for the S–N bond upon binding to Pt [42].

### 3.4. UV-Visible Analysis

**Figure 4:**
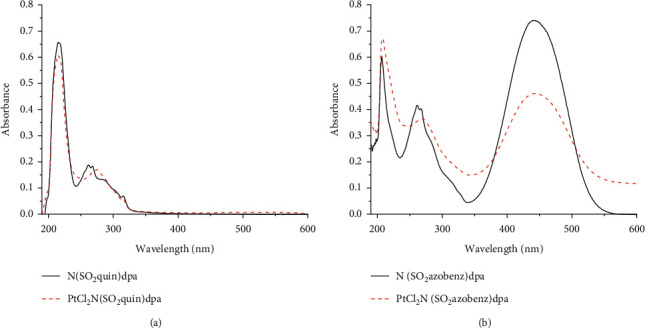
UV-visible spectra of (a) *N*(SO_2_quin)dpa (L1) and PtCl_2_((*N*(SO_2_quin)dpa) (C1), (b) *N*(SO_2_azobenz)dpa (L2) and PtCl_2_ (*N*(SO_2_azobenz)dpa) (C2) in methanol.

The spectra of *N*(SO_2_azobenz)dpa and PtCl_2_(*N*(SO_2_azobenz)dpa) were obtained in methanol (Figure 4). The *N*(SO_2_quin)dpa (L1) shows three absorption peaks at 215, 261, and 315 nm. Qualitatively similar absorptions bands were found for *N*(SO_2_azobenz)dpa (L2) (206, 261, and 442 nm). In the case of PtCl_2_(*N*(SO_2_quin)dpa) (C1) complex, three absorption peaks at 215, 275, and 314 nm were obtained. We have performed TDDFT calculations to rationalize the nature of the absorption bands.

**Figure 5:**
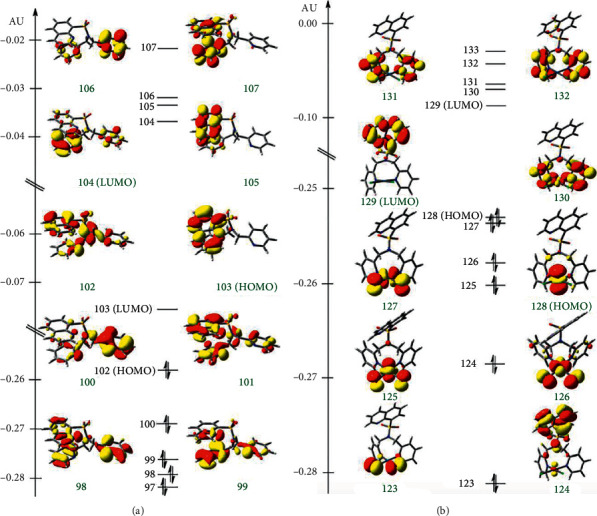
Kohn–Sham frontier orbitals of the ground-state optimized structures of (a) L1 and (b) C1.

Kohn–Sham frontier orbitals of L1 and C1 are shown in Figure 5. HOMO of L1 is delocalized on the quin unit, while LUMO and LUMO + L are delocalized on dpa and quin units, respectively. The calculated HOMO-LUMO gap of L1 4.95 eV (250.6 nm). According to the calculated natural transition orbitals (NTOs) (see Supporting Information), the key excitation of L1 involved quin-based *π⟶π*^*∗*^ excitations 274 nm (*f* = 0.23) and 218 nm (*f* = 1.14), which are consistent with the experimental absorption at 261 and 215 nm. The computed dpa-based *π⟶π*^*∗*^ excitations occur at 229 (*f* = 0.16 nm), 228 (*f* = 0.12), 197 nm (*f* = 0.12), and 196 nm (*f* = 0.20). However, oscillator strengths of the dpa-based *π⟶π*^*∗*^ excitations are smaller than that of the quin-based *π⟶π*^*∗*^ excitations.

In the case of C1, HOMO is localized on Pt and LUMO is delocalized on the quin unit. The calculated HOMO-LUMO gap of C1 is 4.71 eV (273.3 nm), and is relatively smaller than the HOMO-LUMO gap of L1. Computed NTOs suggested that the key excitation involved metal-quin to quin-*π*^*∗*^ charge transfer (i.e., metal-ligand-to-ligand charge transfer, MLLCT) at 228 nm (*f* = 1.15), which is in agreement with the experimental absorption at 215 nm.

### 3.5. Fluorometric Analysis

Emission spectra were obtained for *N*(SO_2_quin)dpa and PtCl_2_((*N*SO_2_quin)dpa) in methanol (Figure S1, Supporting Information). The concentration of the test samples was approximately 0.01 mol/dm^3^. The relevant excitation and emission details are summarized in Table 4. The emission of each system may be attributed to ligand-centered transitions.

**Figure 6:**
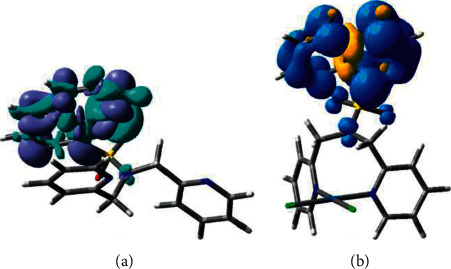
(a) The ground state and lowest singlet excited state electron density difference at the S1 optimized structure of L1. (b) Total spin density distribution of the state of C2 obtained from single-point DFT at the T1 optimized structure of C1.

We have optimized the lowest single excited state (S1) of L1 to obtain the emission wavelength. According to our TDDFT calculations (Supporting Information), the emission of L1 at its lowest single state occurs at 398 nm, which is in agreement with the experimental data (i.e., 418 nm). The electron density difference between the ground and the lowest singlet excited state is shown in Figure 6(a). Moreover, the charge transfer occurs at the quin unit, and therefore, the fluorescence from its lowest singlet excited state can be assigned as the ligand-centered (^1^LC) charge transfer.

In the case of C1, we have optimized the lowest excited singlet (S1) and triplet (T1) states. In C1, the presence of a heavy transition metal (i.e., Pt), the spin-orbit coupling would allow intersystem crossing between the S1 and T1 states. The calculated energy difference between the S1 and T1, 0.51 eV, is relatively large. The computed emission wavelength of the T1 state is 651 nm, which is in agreement with the experimental emission maxima (654 nm). Therefore, the emission of C1 can be assigned to phosphorescence. Starting from the T1 optimized geometry, the total spin density distribution of the triplet state, calculated as the single-point DFT (Figure 6(b)), where the two unpaired electrons are delocalized at quinoline unit. Thus, the phosphorescence of C1 is occurred as the ligand-centered (^3^LC) charge transfer.

Drug-likeness analysis shows that ligands obey the Lipinski's rule of five while the complexes fail only in their size factor (Supporting Information, Figure S2). Probable targets were predicted as voltage-gated potassium channel for L1 and L2 while C1 and C2 targets were predicted as Epidermal growth factor receptor and Vanilloid receptor, respectively (Supporting Information, Figures S3–S6). These predictions may explain the antiproliferative activity shown by the synthesized compounds as the targets given were experimentally shown before to lead in the anticancer drug development path [48–50].

### 3.6. Biological Studies

#### 3.6.1. Emission Activity

**Figure 7:**
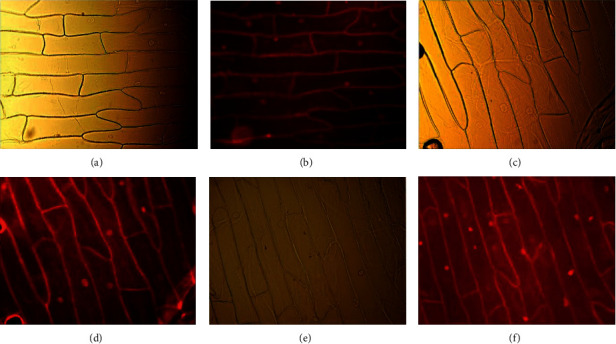
(a) *Allium cepa* cells incubated with (N(SO_2_quin)dpa) in PBS-BSA solution under the optical microscope. (b) Fluorescence micrograph of same cells excited at 550 nm. (c) *Allium cepa* cells incubated with (*N*(SO_2_azobenz)dpa) in PBS-BSA solution under the optical microscope. (d) Fluorescence micrograph of same cells excited at 550 nm. (e) Fluorescence micrograph of *Allium cepa* cells incubated with PtCl_2_ (*N*(SO_2_azobenz)dpa) in PBS-BSA solution, and (f) excited at 550 nm.

In order to study the potential of ligands and platinum complexes as fluorophores, fluorescence microscopy images were obtained with plant cells (*Allium cepa*). The stained *Allium cepa* cells were observed under Olympus BX51 epifluorescence microscope. The obtained micrographs are shown in Figure 7.

According to the obtained results for *N*(SO_2_quin)dpa, *N*(SO_2_azobenz)dpa ligand systems and PtCl_2_(*N*(SO_2_azobenz)dpa) metal complex, the cell wall and the nuclei are prominently stained after incubation with the compound. The compounds show emission under the microscope after binding with cells. This can be due to the alternation of compounds structure, such as increased in conjugation or structural rigidity.

According to fluorescence imaging results on plant cells, these compounds can interact with the nucleus of cells. Due to this reason, we can assume that these compounds may elicit cytotoxicity via genotoxic mechanisms. However, further studies are warranted to explore this mechanism of action.

#### 3.6.2. Anticancer Activity

**Figure 8:**
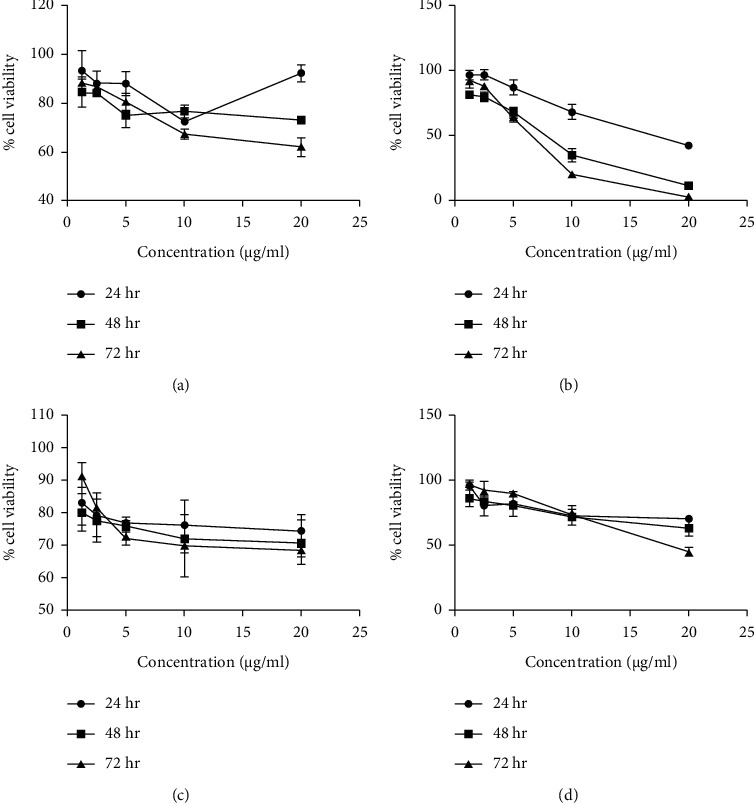
(a) The plot of percentage cell viability vs. the concentration of the compounds of (*N*(SO_2_quin)dpa), (b) PtCl_2_ (*N*(SO_2_quin)dpa), (c) (*N*(SO_2_azobenz)dpa), (d) PtCl_2_(*N*(SO_2_azobenz)dpa).

Sulforhodamine B assay was conducted for the synthesized compounds. NCl–H292 human lung cancer cells were exposed to compounds in a concentration gradient, and cytotoxicity was measured (Figure 8). The half-maximal inhibitory concentration (IC_50_) values were calculated for tested compounds (Table 5), which is a measure of the effectiveness of a tested substance in inhibiting a specific biological activity. Platinum complexes show a significant cytotoxicity compared with their respective ligands. PtCl_2_(*N*(SO_2_quin)dpa elicits ∼6-fold higher cytotoxic activity against NCl–H292 cells compared with *N*(SO_2_quin)dpa at 24 hours. Morphological analysis through phase contrast microscopy shows cell shrinkage, irregular cell shapes and reduction in cell volume which is indicative of apoptotic cell death (Supporting Information, Figures S7–S10). Triggering apoptotic cell death is a significant advantage for a drug lead as it reduces inflammation and damage to peripheral cells.

Cisplatin is a widely used anticancer drug often used to compare the potency of novel drug leads. The website https://www.cancerrxgene.org (accessed on 05/Jul/2022) provides constantly updated data on toxicity of compounds against different cell lines [51] and it reports Cisplatin as having an IC50 of 88.18 *μ*M against NCI–H292. Comparing the IC50 values at 24 h, C1 exhibited higher toxicity than the reported value for Cisplatin, whereas C2 exhibited a 1.7-fold lower activity. This is indicative of the promising potency of the newly synthesized compounds as novel anticancer drug leads. However, L1 ligand-treated cells appear to have started recovering at 72 h. This is not observed with its Pt complex (C1) or the other two compounds (L2 and C2), which further shows their potential to act as anticancer drug leads.

## 4. Conclusions

Two novel platinum complexes as well a novel ligand and a previously studied ligand of which we report a better yield, were synthesized in good yield and characterized using ^1^H NMR, UV-visible, FTIR and fluorescence spectroscopy. The compounds *N*(SO_2_quin)dpa), [PtCl_2_(*N*(SO_2_quin)dpa)], and *N*(SO_2_azobenz)dpa were characterized using single-crystal X-ray diffraction. All compounds were evaluated for their fluorescent imaging ability.

Based on our computational data, we concluded that the quinolene unit-based charge transfer of L1 leads to fluorescence, and quinolene unit-based charge transfer of C1 gives rise to phosphorescence. The fluorescence imaging studies on plant cells revealed that the compounds might be good candidates to be utilized in fluorescence imaging applications, where they clearly associate with the cell nucleus. The metal complexes demonstrated to have high cytotoxicity in comparison with the relevant ligand systems. Reported ligands and their Pt complexes show promising potential to be further studied as potential anticancer drug leads.

## Figures and Tables

**Table 1:** Crystal data and structure refinement for *N*(SO_2_quin)dpa (L1), PtCl_2_(*N*(SO_2_quin)dpa) (C1), and *N*(SO_2_azobenz)dpa (L2).

Crystal data	*N*(SO_2_quin)dpa	PtCl_2_(*N*(SO_2_quin)dpa)	*N*(SO_2_azobenz)dpa
Empirical formula	C_21_H_18_N_4_O_2_S	C_21_H_18_C_l2_N_4_O_2_PtS	C_26_H_26_N_6_O_2_S
CCDC deposition number	2125981	2125982	2125983
Formula weight	390.45	689.50	486.59
Radiation wavelength (Å)	0.71073	0.71073	1.54184
Radiation type	Mo k*α*	Mo k*α*	Cu k*α*
Crystal system	Monoclinic	Triclinic	Monoclinic
Space group	*P*2_1_/*n*	P-1	*P*2_1_/*c*
Unit cell dimensions	—	—	—
*a* (Å)	9.7633 (8)	8.3680 (2)	22.4999 (17)
*b* (Ǻ)	13.3355 (11)	10.4290 (2)	6.0826 (5)
*c* (Ǻ)	13.7589 (11)	13.2551 (3)	17.4488 (13)
*α* (deg)	—	81.6526 (13)	—
*β* (deg)	92.722 (2)	88.2672 (13)	100.973 (6)
*γ* (deg)	—	83.0541 (13)	—
*V* (Å^3^)	1789.4	1136.02 (4)	2344.3 (3)
T (K)	100	100	90
*Z*	4	2	4
Density (Mg m^−3^)	1.449	2.016	1.379
Abs coeff (mm^−1^)	0.21	6.54	1.53
Crystal size (mm)	0.43 × 0.41 × 0.12	0.16 × 0.15 × 0.06	0.17 × 0.04 × 0.01
2*θ*_max_ (deg)	72.7	72.9	128.2
R[*F*^2^ > 2*σ*(F^2^)]	0.036	0.031	0.067
wR(F^2^)	0.101	0.061	0.180
Res. dens (e Ǻ−3)	−0.30,0.59	−1.90, 2.84	−0.39, 0.39
Data/param	8421/253	11007/280	3821/319

**Table 2:** Selected bond lengths/Å and bond angles/° for *N*(SO_2_quin)dpa (L1), PtCl_2_(*N*(SO_2_quin)dpa) (C1), and *N*(SO_2_azobenz)dpa (L2).

	L1		C1		L2	
	Experimental	Calculated	Experimental	Calculated	Experimental	Calculated
S1–O1	1.4338 (7)	1.44	1.428 (2)	1.43	1.432 (3)	1.44
S1–O2	1.4382 (6)	1.44	1.433 (2)	1.44	1.435 (3)	1.44
S1–N2	1.6225 (7)	1.62	1.632(2)	1.63	1.620(4)	1.63
S1–C13	1.7682 (8)	1.77	1.763 (3)	1.77	1.765 (4)	1.76
N1–C1	1.3398 (11)	1.33	1.347 (3)	1.34	1.347 (6)	1.33
N1–C5	1.3408 (11)	1.33	1.352 (3)	1.34	1.345 (5)	1.33
N2–C6	1.4581 (10)	1.45	1.462 (3)	1.46	1.469 (5)	1.45
N2–C7	1.4584 (10)	1.46	1.469 (3)	1.46	1.461 (5)	1.45
N3–C12	1.3412 (12)	1.33	1.345 (3)	1.34	1.345 (6)	1.33
N3–C8	1.3415 (11)	1.33	1.351 (3)	1.34	1.350 (5)	1.33
Pt–N1	—	—	2.017 (2)	2.03	—	—
Pt–N3	—	—	2.023 (2)	2.03	—	—
Pt–Cl2	—	—	2.2942 (6)	2.32	—	—
Pt–Cl1	—	—	2.2962 (6)	2.32	—	—
O1–S1–O2	118.94 (4)	118.95	118.78 (13)	118.78	119.19 (18)	119.18
O1–S1–N2	106.70 (4)	106.70	106.81 (12)	106.82	108.12 (19)	108.11
O2–S1–N2	108.44 (4)	108.44	106.99 (12)	106.98	106.19 (18)	106.21
C6–N2–C7	119.68 (6)	119.68	120.80 (2)	120.71	118.10 (3)	118.07
C6–N2–S1	119.70 (5)	119.70	119.80 (17)	119.82	118.90 (3)	118.97
C7–N2–S1	119.58 (5)	119.59	117.38 (17)	117.40	120.60 (3)	120.62
N1–C5–C6	113.19 (7)	113.19	118.50 (2)	118.51	116.20 (4)	116.20
C1–N1–C5	117.49 (8)	117.49	119.30 (2)	119.21	116.80 (4)	116.85
C12–N3–C8	117.43 (7)	117.43	119.80 (2)	119.82	116.50 (4)	116.56
N1–Pt1–N3	—	—	88.66 (8)	88.66	—	—
N3–Pt1–Cl2	—	—	91.02 (6)	91.02	—	—
N1–Pt1–Cl1	—	—	88.18 (6)	88.18	—	—

**Table 3:** Comparison of selected ^1^H NMR shifts of compounds in DMSO-d_6_.

Product	H6/6′	H5/5′	H4/4′	H3/3′	CH_2_
*N*(SO_2_quin)dpa	8.21	7.07	7.51	7.11	4.81
PtCl_2_(*N*(SO_2_quin)dpa)	9.26	7.52	7.97	7.70	5.95, 5.61
*N*(SO_2_azobenz)dpa	8.36	7.19	7.67	7.28	4.58
PtCl_2_(*N*(SO_2_azobenz)dpa)	9.26	7.52	7.97	7.70	6.04, 5.28

**Table 4:** Excitation and emission wavelengths of compounds in methanol. The excitation wavelength was 320 nm.

Compound	Emission max. wavelength/nm
*N*(SO_2_quin)dpa (L1)	418
PtCl_2_(*N*(SO_2_quin)dpa) (C1)	420
*N*(SO_2_azobenz)dpa (L2)	441
PtCl_2_(*N*(SO_2_azobenz)dpa) (C2)	423

**Table 5:** IC_50_ values reported for ligands and the complexes at 24, 48, and 72 h incubation period.

Sample	IC_50_ values in *μ*g/mL (mM)		
	24 h	48 h	72 h
*N*(SO_2_quin)dpa	225 (0.58 mM)	31.82 (0.08 mM)	36.49 (0.09 mM)
PtCl_2_(*N*(SO_2_quin)dpa)	37.83 (0.06 mM)	15.94 (0.02 mM)	9.01 (0.01 mM)
*N*(SO_2_azobenz)dpa	612.4 (1.26 mM)	17.88 (0.04 mM)	13.95 (0.03 mM)
PtCl_2_(*N*(SO_2_azobenz)dpa)	112.8 (0.15 mM)	26.21 (0.03 mM)	12.13 (0.02 mM)

## Data Availability

The data used to support the findings of this study are included within the article.

## Supplementary Materials

Supplementary Materials Supporting information for this article is available with the submitted manuscript; tabulated IR peaks of ligands and their metal complexes, emission spectra of L1, L2, C1, and C2 obtained in methanol, TDDFT results, SwissADME predictions of pharmacokinetic properties and predicted targets of L1, L2, C1, and C2, and morphology of MRC-5 cells and human lung cancer cells (NCl–H292) after 24, 48, and 72 h incubating with L1, C1, L2, and C2 in increasing concentrations are available.
