# Supplementary material for: Biological Evaluation of Platinum(II) Sulfonamido Complexes: Synthesis, Characterization, Cytotoxicity, and Biological Imaging
Source: Bioinorg Chem Appl. 2022 Sep 13;2022:7821284. doi: 10.1155/2022/7821284 (PMC9489406; doi:10.1155/2022/7821284)
Supplement: Supplementary Materials — Supporting information for this article is available with the submitted manuscript; tabulated IR peaks of ligands and their metal complexes, emission spectra of L1, L2, C1, and C2 obtained in methanol, TDDFT results, SwissADME predictions of pharmacokinetic properties and predicted targets of L1, L2, C1, and C2, and morphology of MRC-5 cells and human lung cancer cells (NCl–H292) after 24, 48, and 72 h incubating with L1, C1, L2, and C2 in increasing concentrations are available. [file 7821284.f1.docx]

**Biological evaluation of platinum(II) sulfonamido complexes: synthesis, characterization, cytotoxicity and biological imaging**

Supporting Information

# Charini Maladeniya^1^, Taniya Darshani^1^, Sameera R. Samarakoon^2^, Frank R. Fronczek^3^, W. M. C. Sameera^4^, Inoka C. Perera^5^, Theshini Perera*^1^

^1^ Department of Chemistry, University of Sri Jayewardenepura, Sri Lanka. *theshi@sjp.ac.lk

^2^Institute of Biochemistry, Molecular Biology and Biotechnology, University of Colombo, Sri Lanka.

^3^ Department of Chemistry, Louisiana State University, Baton Rouge, LA, USA.

^4^Institute of Low Temperature Science, Hokkaido University, N19-W8, Kita ku, Sapporo, Hokkaido 060-0819, Japan.

^5^Department of Zoology and Environmental Science, University of Colombo, Sri Lanka.

Section Page

1. Characteristic IR peaks of the ligands and their metal complexes in cm^-1^ 2

2. Emission spectra of L1, L2, C1 and C2 obtained in methanol 3

3. TDDFT results 4

4. SwissADME predictions of pharmacokinetic properties of L1, L2, C1 and C2. 20

5. Predicted Targets of L1, L2, C1, and C2. 22

6. Morphology of MRC-5 cells after 24, 48 and 72hr incubating with L1 and C1 in increasing

concentrations 26

7. Morphology of MRC-5 cells after 24, 48 and 72hr incubating with L2 and C2 in increasing

concentrations 27

8. Morphology of human lung cancer cells (NCl-H292) after 24, 48 and 72hr incubating with L1

and C1 in increasing concentrations 28

9. Morphology of human lung cancer cells (NCl-H292) after 24, 48 and 72hr incubating with L2

and C2 in increasing concentrations 29

Table S1. The characteristic IR peaks of the ligands and their metal complexes in cm^-1^

| **Compound** | **νS-N** | **ν_as_(SO_2_)** | **ν_s_(SO_2_)** |
| --- | --- | --- | --- |
| *N*(SO_2_quin)dpa**(L1)** | 930 | 1328 | 1140 |
| PtCl_2_(*N*(SO_2_quin)dpa) **(C1)** | 894 | 1331 | 1147 |
| *N*(SO_2_azobenz)dpa**(L2)** | 917 | 1330 | 1141 |
| PtCl_2_(*N*(SO_2_azobenz)dpa) **(C2)** | 879 | 1326 | 1136 |








**Figure S1;** Emission spectra (top) and normalized emission spectra (bottom) of L1, L2, C1 and C2 *N*(SO_2_quin)dpa (L1), PtCl_2_(*N*(SO_2_quin)dpa) (C1), *N*(SO_2_azobenz)dpa (L2) and PtCl_2_(*N*(SO_2_ azobenz)dpa) (C2)obtained in methanol

**TDDFT Results - Calculated vertical excitation energies and the natural transition orbitals [Highest occupied transition orbitals (HOTO) and lowest unoccupied natural transition orbitals (LUTO)] of the key excitations of L1 and C1.**

**L1**

Excited State 1: Singlet 4.4566 eV 278.20 nm f=0.0195 <S**2>=0.000

96 ->103 0.43328

96 ->109 -0.10105

97 ->103 0.37612

99 ->103 -0.11363

101 ->103 -0.16778

102 ->103 0.24925

Excited State 2: Singlet 4.5330 eV 273.52 nm f=0.2258 <S**2>=0.000

96 ->103 -0.15258

98 ->103 0.16460

99 ->103 -0.29235

101 ->103 0.24352

102 ->103 0.50689


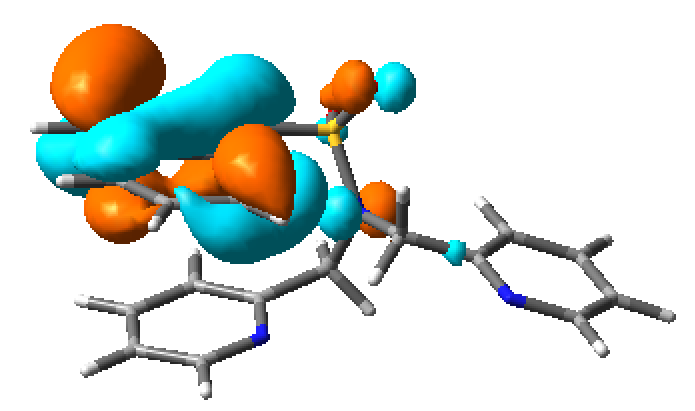

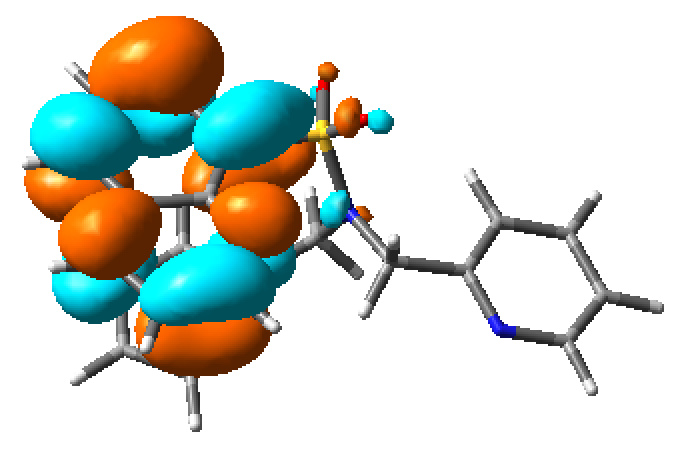


**HOTO LUTO**

Excited State 3: Singlet 4.6280 eV 267.90 nm f=0.1841 <S**2>=0.000

99 ->103 0.48081

100 ->103 0.21599

101 ->103 0.18328

102 ->103 0.29958

102 ->104 -0.13694

102 ->105 0.18509

Excited State 4: Singlet 5.0412 eV 245.94 nm f=0.0086 <S**2>=0.000

95 ->104 0.21099

95 ->105 0.13505

95 ->106 -0.10253

96 ->104 0.22876

96 ->105 0.14791

96 ->106 -0.12692

98 ->104 0.14138

100 ->104 0.22465

100 ->105 0.13413

100 ->106 -0.10292

101 ->104 0.26671

101 ->105 0.15470

101 ->106 -0.10253

102 ->104 -0.21751

102 ->105 -0.14275

Excited State 5: Singlet 5.1753 eV 239.57 nm f=0.0055 <S**2>=0.000

95 ->106 0.11855

96 ->104 -0.11945

96 ->106 -0.26236

97 ->104 0.18294

97 ->105 0.11773

97 ->106 0.45889

99 ->106 -0.11301

100 ->106 0.22913

102 ->106 -0.15559

Excited State 6: Singlet 5.3634 eV 231.17 nm f=0.0389 <S**2>=0.000

95 ->103 -0.14397

96 ->103 0.20307

99 ->103 -0.14307

100 ->103 0.24186

100 ->106 -0.10944

101 ->103 0.45777

102 ->103 -0.21638

Excited State 7: Singlet 5.4179 eV 228.84 nm f=0.1574 <S**2>=0.000

93 ->107 -0.10416

94 ->108 0.15422

97 ->106 -0.10231

98 ->104 0.20480

98 ->105 0.14270

98 ->106 -0.22112

99 ->106 -0.11725

100 ->103 0.13037

100 ->104 0.13558

100 ->106 0.24866

100 ->107 0.10597

101 ->104 -0.23925

101 ->105 -0.17119

101 ->106 -0.18821

102 ->106 0.12403


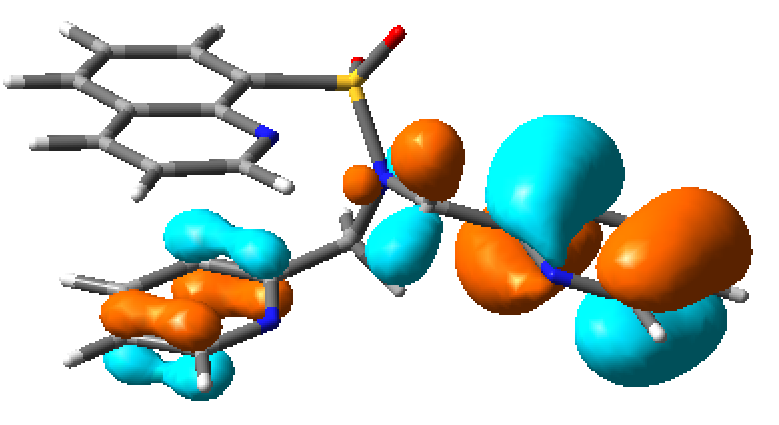

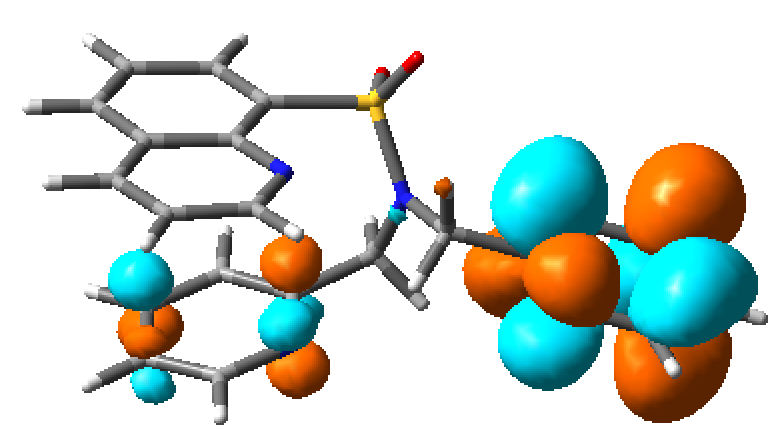


**HOTO LUTO**

Excited State 8: Singlet 5.4401 eV 227.91 nm f=0.1183 <S**2>=0.000

93 ->107 -0.13842

94 ->108 -0.14602

98 ->103 0.16889

98 ->104 0.30417

98 ->105 0.21175

99 ->104 0.12236

100 ->106 -0.27040

101 ->106 0.31010


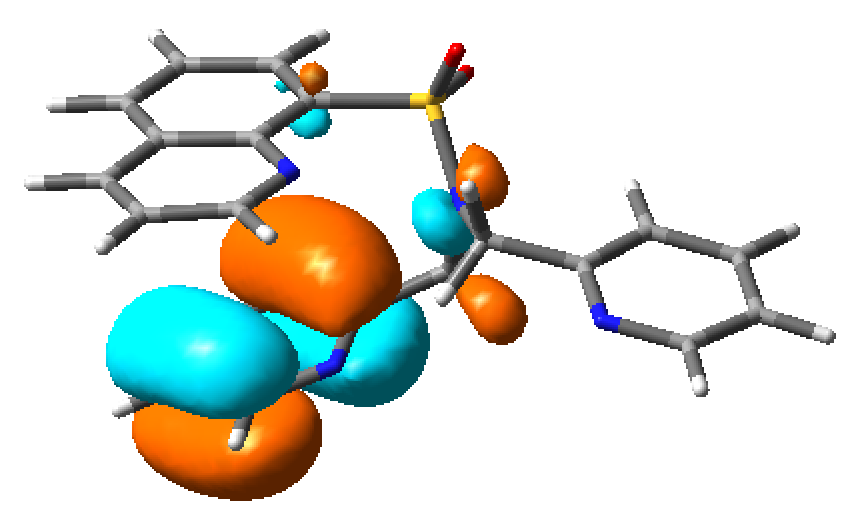

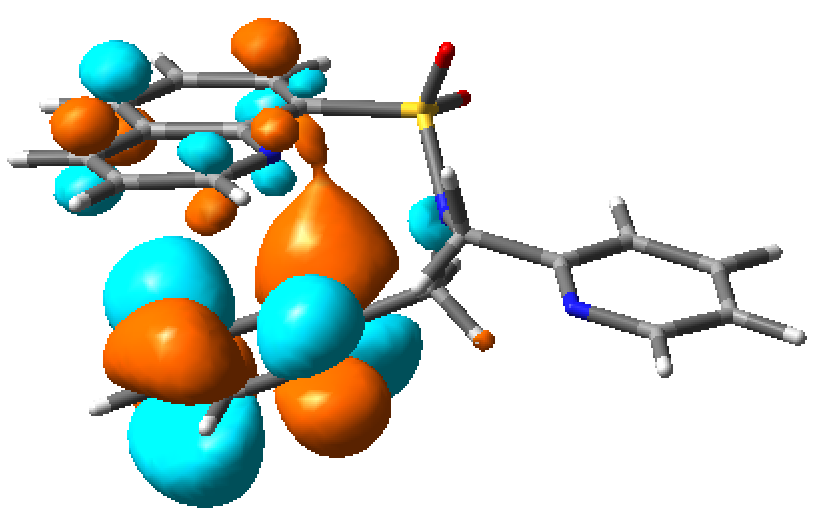


**HOTO LUTO**

Excited State 9: Singlet 5.5065 eV 225.16 nm f=0.0051 <S**2>=0.000

95 ->107 0.21393

96 ->107 0.26139

96 ->109 0.11724

98 ->104 -0.12249

98 ->107 0.14658

100 ->107 0.24468

101 ->105 0.10339

101 ->107 0.28113

101 ->109 0.10266

102 ->107 -0.25868

Excited State 10: Singlet 5.5809 eV 222.16 nm f=0.0020 <S**2>=0.000

96 ->104 -0.24944

96 ->105 0.38169

97 ->104 -0.20770

97 ->105 0.31898

98 ->103 0.15690

98 ->105 -0.11758

101 ->104 0.12392

101 ->105 -0.16001

Excited State 11: Singlet 5.5865 eV 221.93 nm f=0.0624 <S**2>=0.000

95 ->103 -0.10699

98 ->103 0.53889

98 ->104 -0.14200

99 ->103 0.12441

100 ->103 0.20528

101 ->103 -0.11897

Excited State 12: Singlet 5.6558 eV 219.22 nm f=0.0038 <S**2>=0.000

95 ->108 0.11371

96 ->108 -0.29292

97 ->108 0.50633

99 ->108 -0.12415

100 ->108 0.24897

101 ->108 0.10443

102 ->108 -0.17784

Excited State 13: Singlet 5.6818 eV 218.21 nm f=1.1436 <S**2>=0.000

98 ->103 0.19876

99 ->103 -0.26957

101 ->104 -0.15546

101 ->105 0.21189

102 ->104 -0.29483

102 ->105 0.43372


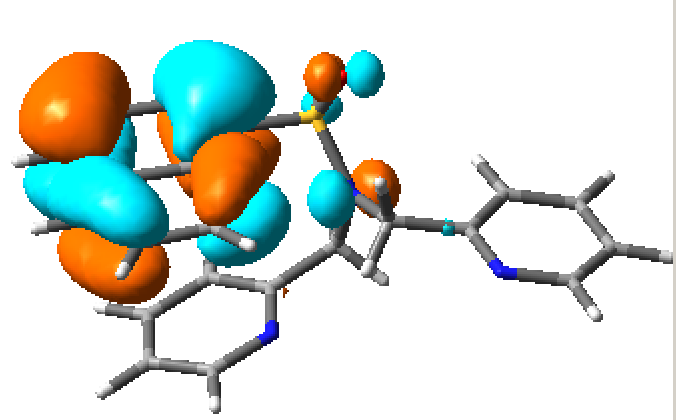

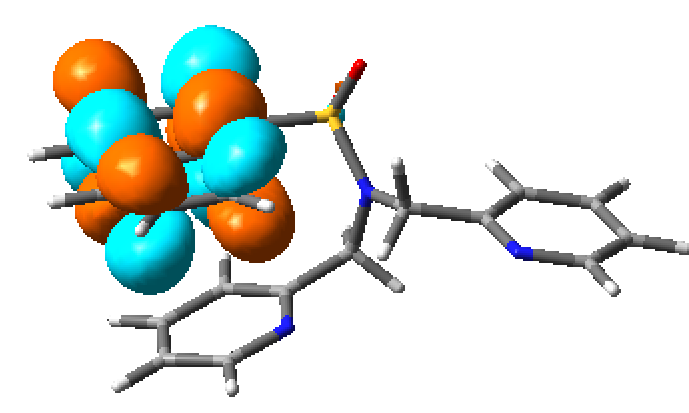


**HOTO LUTO**

Excited State 14: Singlet 6.0970 eV 203.35 nm f=0.1321 <S**2>=0.000

95 ->103 0.41414

96 ->103 0.24853

98 ->103 0.10540

99 ->104 -0.11108

99 ->105 0.17269

101 ->103 0.11872

101 ->107 -0.13862

102 ->107 -0.18264

102 ->109 0.25728

Excited State 15: Singlet 6.1460 eV 201.73 nm f=0.0361 <S**2>=0.000

95 ->104 0.21593

95 ->105 0.11424

98 ->104 0.14099

98 ->107 -0.19923

101 ->107 0.13628

101 ->108 -0.10993

102 ->104 0.40241

102 ->105 0.26581

102 ->106 -0.16316

Excited State 16: Singlet 6.1899 eV 200.30 nm f=0.0148 <S**2>=0.000

95 ->103 0.25997

96 ->103 0.14953

98 ->109 -0.11429

99 ->107 -0.10002

101 ->107 0.24016

101 ->109 -0.12184

102 ->104 -0.15706

102 ->105 -0.12220

102 ->107 0.33560

102 ->109 -0.24395

Excited State 17: Singlet 6.2117 eV 199.60 nm f=0.3033 <S**2>=0.000

91 ->103 -0.17259

92 ->103 0.16815

95 ->103 -0.11764

96 ->103 -0.15215

96 ->107 0.12762

96 ->109 -0.17607

97 ->109 -0.15688

98 ->104 0.10760

98 ->105 -0.10998

99 ->104 -0.23149

99 ->105 0.33702

99 ->107 -0.11234

100 ->105 0.11130

101 ->109 0.12910

Excited State 18: Singlet 6.2446 eV 198.55 nm f=0.1110 <S**2>=0.000

93 ->106 0.10697

94 ->106 0.21339

98 ->108 0.12422

99 ->108 0.10855

100 ->108 -0.35286

101 ->108 0.37218

102 ->106 -0.10028

102 ->108 -0.21267


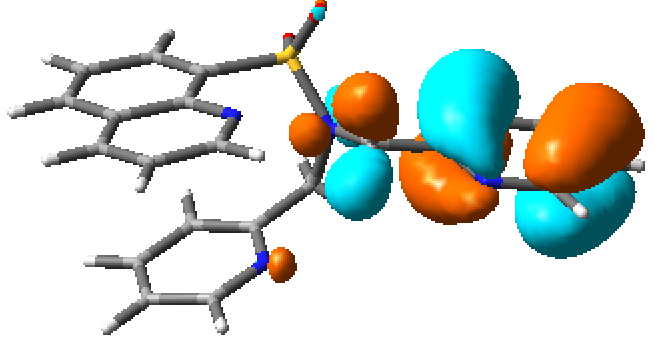

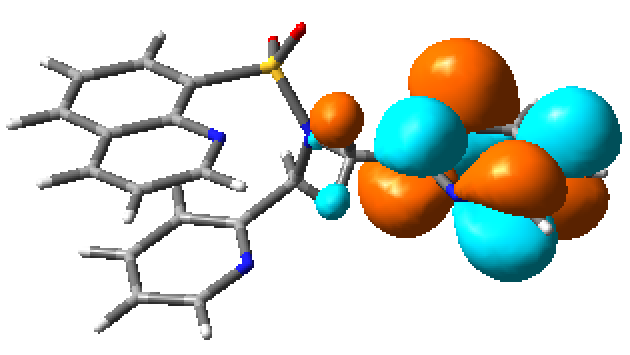


**HOTO LUTO**

Excited State 19: Singlet 6.2816 eV 197.38 nm f=0.1213 <S**2>=0.000

93 ->104 0.18236

93 ->105 0.11096

98 ->107 0.40709

101 ->107 -0.12936

101 ->109 -0.18512

102 ->104 0.13518

102 ->105 0.11409

102 ->106 -0.10324

102 ->107 0.15650

102 ->109 -0.26851


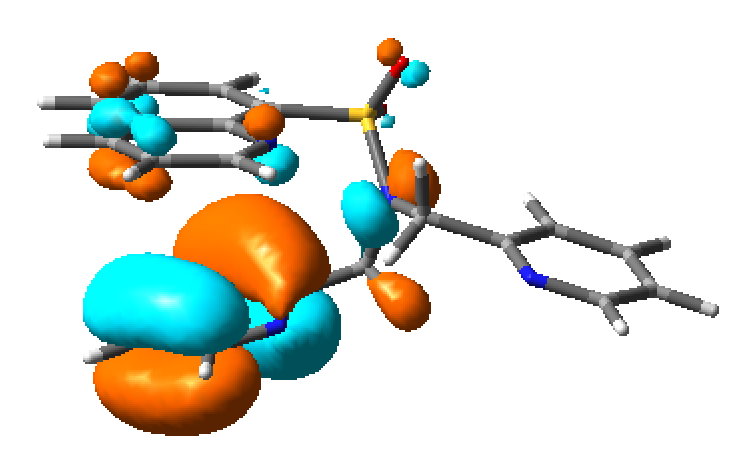

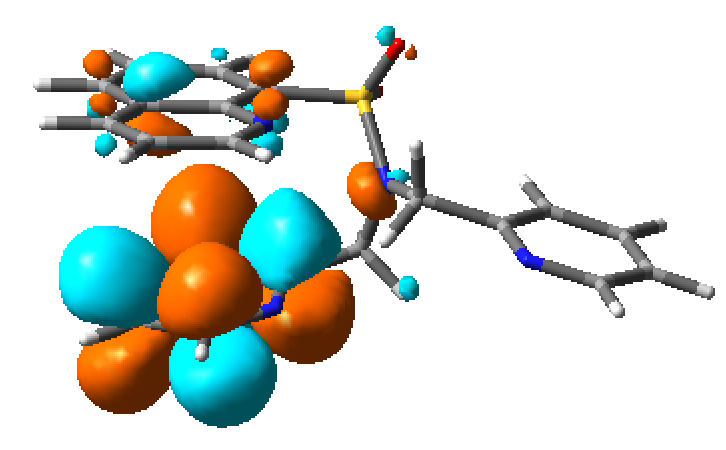


**HOTO LUTO**

Excited State 20: Singlet 6.3115 eV 196.44 nm f=0.1950 <S**2>=0.000

91 ->103 0.12039

92 ->103 -0.11950

95 ->103 -0.22119

96 ->107 -0.14154

96 ->109 0.18739

97 ->107 -0.10902

97 ->109 0.14907

99 ->104 -0.21987

99 ->105 0.27904

99 ->107 0.11169

99 ->109 -0.20480

100 ->104 -0.11531

100 ->105 0.14605

**
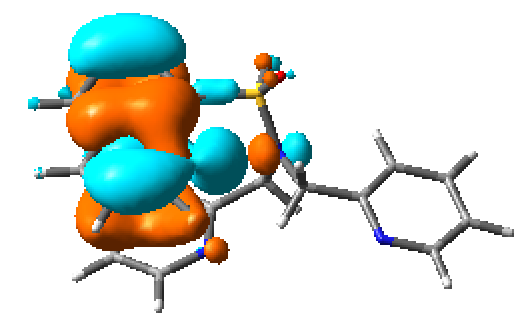
** **
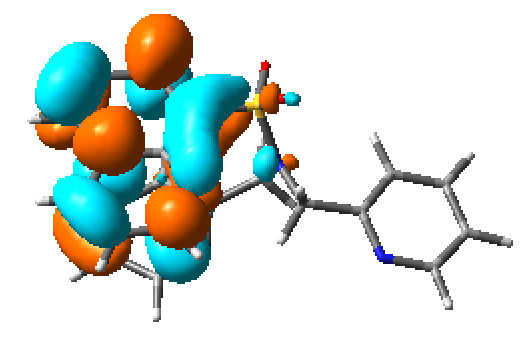
**

**HOTO LUTO**

**C1**

Excited State 1: Singlet 3.5668 eV 347.61 nm f=0.0002 <S**2>=0.000

126 -> 133 0.61194

127 -> 133 0.27873

128 -> 133 0.16254

Excited State 2: Singlet 3.7702 eV 328.85 nm f=0.3727 <S**2>=0.000

126 -> 129 -0.13517

128 -> 129 0.67711

Excited State 3: Singlet 3.8153 eV 324.96 nm f=0.0021 <S**2>=0.000

105 -> 133 -0.13263

125 -> 133 0.66401

Excited State 4: Singlet 3.8470 eV 322.28 nm f=0.0001 <S**2>=0.000

115 -> 133 -0.17500

126 -> 133 -0.27831

127 -> 133 0.60889

Excited State 5: Singlet 3.9514 eV 313.77 nm f=0.0003 <S**2>=0.000

113 -> 133 0.15479

124 -> 133 0.66205

Excited State 6: Singlet 4.1032 eV 302.16 nm f=0.0007 <S**2>=0.000

118 -> 129 0.14857

119 -> 129 -0.23502

120 -> 129 -0.40375

121 -> 129 -0.14842

122 -> 129 0.44730

Excited State 7: Singlet 4.3646 eV 284.07 nm f=0.1675 <S**2>=0.000

121 -> 129 0.60519

122 -> 129 0.15878

128 -> 134 0.28623

Excited State 8: Singlet 4.6700 eV 265.49 nm f=0.0011 <S**2>=0.000

126 -> 130 0.60800

127 -> 130 0.28035

128 -> 130 0.17329

Excited State 9: Singlet 4.8858 eV 253.77 nm f=0.0001 <S**2>=0.000

126 -> 131 0.60750

127 -> 131 0.27476

128 -> 131 0.17183

Excited State 10: Singlet 5.0042 eV 247.76 nm f=0.0026 <S**2>=0.000

115 -> 130 -0.12933

126 -> 130 -0.28402

127 -> 130 0.60475

Excited State 11: Singlet 5.0514 eV 245.45 nm f=0.0246 <S**2>=0.000

113 -> 130 0.10488

124 -> 130 0.58947

125 -> 130 -0.25555

127 -> 130 0.10006

127 -> 131 -0.14457

128 -> 130 -0.12086

Excited State 12: Singlet 5.1856 eV 239.09 nm f=0.1568 <S**2>=0.000

123 -> 133 0.10044

124 -> 130 0.26860

125 -> 130 0.60147

Excited State 13: Singlet 5.2259 eV 237.25 nm f=0.0174 <S**2>=0.000

115 -> 131 -0.13349

124 -> 130 0.12510

125 -> 131 -0.12841

126 -> 131 -0.24551

127 -> 131 0.58070

Excited State 14: Singlet 5.2357 eV 236.81 nm f=0.1503 <S**2>=0.000

120 -> 129 -0.20586

122 -> 129 -0.18695

124 -> 131 -0.28622

125 -> 131 0.47452

128 -> 131 0.11265

Excited State 15: Singlet 5.2562 eV 235.88 nm f=0.0840 <S**2>=0.000

117 -> 129 -0.10541

118 -> 131 0.10070

119 -> 129 0.10557

120 -> 129 0.33289

122 -> 129 0.30759

124 -> 129 -0.10063

124 -> 131 -0.17946

125 -> 131 0.22615

126 -> 129 0.10498

127 -> 131 0.15990

128 -> 134 -0.11022

Excited State 16: Singlet 5.2894 eV 234.40 nm f=0.0176 <S**2>=0.000

118 -> 131 -0.21472

119 -> 130 -0.27906

120 -> 130 0.13090

122 -> 131 0.18982

123 -> 130 0.12653

124 -> 131 0.33224

125 -> 131 0.32903

125 -> 135 0.11819

Excited State 17: Singlet 5.3255 eV 232.81 nm f=0.1629 <S**2>=0.000

116 -> 135 -0.12125

117 -> 132 0.13307

118 -> 130 0.30585

119 -> 130 0.13447

119 -> 131 0.33374

120 -> 129 0.12239

120 -> 130 -0.13888

120 -> 131 -0.15100

122 -> 129 0.10023

122 -> 130 -0.21704

125 -> 132 0.24230

Excited State 18: Singlet 5.3455 eV 231.94 nm f=0.0743 <S**2>=0.000

118 -> 131 0.22750

119 -> 130 0.27267

120 -> 130 -0.11851

122 -> 131 -0.17471

124 -> 131 0.45746

125 -> 131 0.19088

128 -> 131 -0.11161

Excited State 19: Singlet 5.4431 eV 227.78 nm f=1.1457 <S**2>=0.000

120 -> 129 0.10838

121 -> 129 -0.27350

126 -> 129 0.12275

126 -> 132 0.14116

126 -> 134 -0.10139

128 -> 134 0.55323


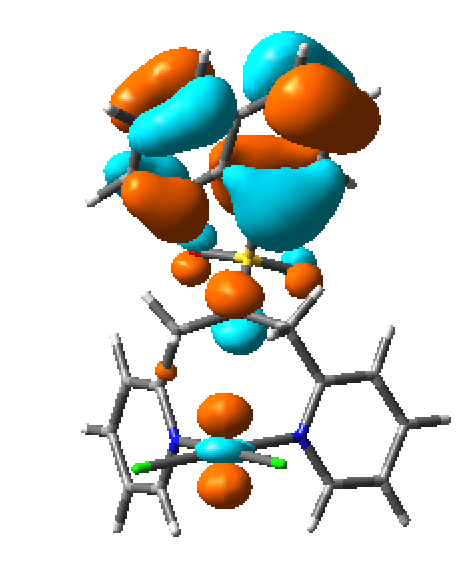

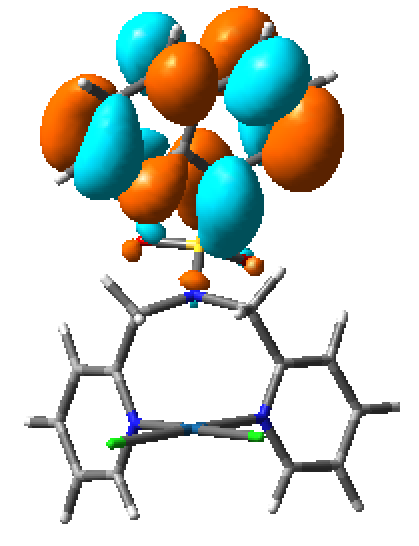


**HOTO LUTO**

Excited State 20: Singlet 5.4659 eV 226.83 nm f=0.0721 <S**2>=0.000

120 -> 129 -0.10100

126 -> 129 0.30549

126 -> 132 0.48212

127 -> 129 0.13875

127 -> 132 0.22006

128 -> 132 0.16198

128 -> 134 -0.11735

**Cartesian coordinates of the optimized structures**

**L1**

**Ground state optimized structure**

S 0.34964900 1.84583800 0.18446900

O 0.47882500 2.82980400 -0.85769600

O 0.85629000 2.15821000 1.49074000

N -0.32361300 -2.05098600 -1.13384000

N 1.09693600 0.49973900 -0.31950700

N 3.93470600 -1.44084300 0.86198600

N -1.00158600 -0.34282100 1.77610500

C -1.41120700 -2.81394400 -1.12429600

H -1.32711900 -3.76399000 -0.60435500

C -2.60402200 -2.45487900 -1.73362900

H -3.46123200 -3.11473100 -1.68767000

C -2.65987200 -1.24278700 -2.40059700

H -3.56881700 -0.92269400 -2.89616000

C -1.53269400 -0.43845500 -2.41224600

H -1.54001200 0.52338200 -2.90985400

C -0.38912500 -0.87124900 -1.75251400

C 0.83181200 0.00738700 -1.66966100

H 1.71695800 -0.55264100 -1.97983800

H 0.73125000 0.86161400 -2.33792600

C 1.80942000 -0.36764100 0.59649000

H 1.41206700 -1.38032200 0.52027000

H 1.61726800 -0.02758000 1.61268700

C 3.29460700 -0.39150400 0.34525000

C 3.95322800 0.61763900 -0.34642300

H 3.39377800 1.45109800 -0.75180900

C 5.32676800 0.53298100 -0.50327200

H 5.86563700 1.30525900 -1.04007400

C 5.99759300 -0.55216900 0.03660000

H 7.07022800 -0.66161600 -0.06217800

C 5.25368200 -1.50813900 0.71089500

H 5.74322500 -2.37472700 1.14668300

C -1.38319700 1.48987800 0.28161300

C -2.24389100 2.26497200 -0.45128700

H -1.84641500 3.07795000 -1.04396400

C -3.62291700 1.99507000 -0.45809800

H -4.28293700 2.62191500 -1.04454400

C -4.11880500 0.93881600 0.25349600

H -5.17859700 0.70953400 0.23975700

C -3.25788200 0.12325200 1.01764300

C -1.86594200 0.40120100 1.05453300

C -1.47337700 -1.36264500 2.44839300

H -0.74988600 -1.94396600 3.01418900

C -2.83079100 -1.73229200 2.47090300

H -3.14680900 -2.59178400 3.04809600

C -3.72170900 -0.98317000 1.75679000

H -4.77905000 -1.22377800 1.74412500

**Lowest singlet state optimized structure**

S 1.28099500 -0.75101800 0.06486700

O 0.40853600 0.32951200 -0.30201800

O 1.68373200 -1.67992600 -0.95485000

N 1.45185100 -1.94058400 4.09810600

N 0.58077000 -1.64348400 1.23497000

N -1.06158400 -4.91026600 1.08261200

N 3.47781500 -2.18506700 1.56419500

C 2.37910100 -1.85220800 5.04588500

H 2.71555000 -2.78879300 5.47735400

C 2.91069900 -0.64911500 5.48342600

H 3.66935500 -0.63681600 6.25385300

C 2.44379300 0.52084700 4.91068000

H 2.83061200 1.48293000 5.22075900

C 1.48109300 0.43810900 3.91917800

H 1.10858600 1.32859700 3.43086300

C 1.02099900 -0.81281600 3.53269200

C 0.02568600 -0.96061500 2.40419300

H -0.83233000 -1.54408000 2.74173500

H -0.34742400 0.01571200 2.09870200

C 0.50093000 -3.09306800 1.17575500

H 0.78735900 -3.49401700 2.14828400

H 1.22525300 -3.45600800 0.44793600

C -0.86751000 -3.62085700 0.80538200

C -1.83772200 -2.83291000 0.20185800

H -1.64284700 -1.78952200 -0.00632300

C -3.05451400 -3.40788100 -0.12564700

H -3.83008400 -2.81617100 -0.59497300

C -3.26025500 -4.74759200 0.15561700

H -4.19409400 -5.23839000 -0.08158600

C -2.23092100 -5.45343400 0.75798500

H -2.35389300 -6.50496000 0.99532000

C 2.67679100 -0.03518300 0.82024300

C 2.88883200 1.35167900 0.82556500

H 2.17758600 1.98932800 0.32025000

C 3.97595500 1.87879900 1.47482700

H 4.13584300 2.94819800 1.47614400

C 4.87789600 1.04268700 2.14675300

H 5.72190800 1.46962600 2.67548800

C 4.72461300 -0.33183400 2.17805400

C 3.57886400 -0.88738400 1.47628600

C 4.18855800 -3.11485900 2.18779500

H 3.89937700 -4.15194600 2.12734700

C 5.29723800 -2.60996000 2.87679400

H 5.92726400 -3.31376600 3.40417200

C 5.56220300 -1.26375500 2.86846900

H 6.41713400 -0.87733800 3.40769800

**C1**

**Ground state optimized structure**

Pt 5.39388200 7.30448800 8.56917200

Cl 3.34874900 7.04645600 9.62739500

Cl 5.97200200 5.08979600 8.94209700

S 8.60627900 11.06925400 9.98659900

O 7.80819700 12.24791900 10.16087500

O 9.54045400 11.01570000 8.89597400

N 4.91094400 9.24113100 8.17773100

N 7.58453800 9.81244300 9.82761300

N 7.16061500 7.55905800 7.59383100

N 7.58120000 10.65780200 12.80027700

C 4.15822000 9.48640000 7.09717700

H 3.85285800 8.62126000 6.52509700

C 3.78614000 10.76295600 6.73431400

H 3.17630800 10.90889300 5.85304900

C 4.20275200 11.82540800 7.51862200

H 3.92649600 12.84181700 7.26673300

C 4.97755700 11.56450800 8.63354800

H 5.33401800 12.36200900 9.27137800

C 5.32538200 10.26014800 8.94771100

C 6.17320800 9.96514100 10.15124100

H 6.07572400 10.77105700 10.87392700

H 5.83155000 9.04478000 10.62779700

C 8.17707000 8.49789500 9.62004800

H 7.59834300 7.76792700 10.18900300

H 9.18903500 8.49870300 10.03038000

C 8.25365900 8.07448500 8.17969400

C 9.43625100 8.20409000 7.46950700

H 10.29845700 8.62510000 7.96794100

C 9.49621800 7.81300800 6.14493700

H 10.41735900 7.91176400 5.58418800

C 8.35772500 7.29352800 5.55308200

H 8.34773000 6.97039100 4.52097400

C 7.21157000 7.17921900 6.31007200

H 6.29959600 6.76733800 5.90061600

C 9.55691900 10.76272100 11.44657400

C 10.92117100 10.69191300 11.32696300

H 11.37141300 10.80237000 10.34948700

C 11.72875100 10.48170700 12.45802500

H 12.80384500 10.43265200 12.33999900

C 11.15752000 10.33974900 13.69142500

H 11.77153900 10.17609600 14.57006000

C 9.75558500 10.39683100 13.84403900

C 8.92755700 10.60985600 12.71064600

C 7.03278000 10.50448800 13.98027500

H 5.94733400 10.54430400 14.01651500

C 7.76027100 10.29603300 15.16632800

H 7.23716200 10.17985500 16.10673700

C 9.12286500 10.24237600 15.09419000

H 9.72830100 10.08067600 15.97913500

**Lowest singlet state optimized structure**

Pt 5.20366000 7.11995800 8.65605800

Cl 2.86757200 6.81848000 9.47748400

Cl 5.85875500 4.76848400 8.93585100

S 8.61330300 11.16834000 10.00517600

O 7.82060800 12.34460500 10.21975500

O 9.53961100 11.14892900 8.90675000

N 4.77286200 9.26145600 8.26661400

N 7.58726400 9.91733800 9.81737700

N 7.21072000 7.54634300 7.66996600

N 7.60435600 10.63624700 12.80081400

C 3.99712800 9.47750400 7.20132800

H 3.57235200 8.59604600 6.73593900

C 3.73394300 10.74064400 6.71252200

H 3.09850100 10.86024800 5.84514800

C 4.29460900 11.82800000 7.36228900

H 4.11076000 12.83712200 7.01412000

C 5.08742700 11.60245600 8.47202900

H 5.54363600 12.42108100 9.01334800

C 5.31566600 10.30342100 8.90685300

C 6.17301900 10.07070900 10.12338100

H 6.06635200 10.91386400 10.80128600

H 5.84061900 9.18600400 10.66924200

C 8.18450500 8.59757600 9.65839800

H 7.61524800 7.88921200 10.26352100

H 9.19319700 8.61698500 10.07640100

C 8.27543800 8.12460000 8.23242900

C 9.45567100 8.29226400 7.52110500

H 10.30080900 8.76196400 8.00603200

C 9.52893900 7.87518100 6.20487500

H 10.44374300 8.00127500 5.63877600

C 8.41226300 7.29489600 5.62702100

H 8.41367600 6.95305900 4.60053800

C 7.27778700 7.14676200 6.39921700

H 6.38366500 6.68543300 5.99573700

C 9.57380800 10.81012800 11.44649800

C 10.93764300 10.74883400 11.31687400

H 11.38235200 10.90237200 10.34267300

C 11.75123100 10.48927600 12.43311400

H 12.82582300 10.44794800 12.30773900

C 11.18606000 10.28781500 13.66112300

H 11.80460700 10.08395900 14.52811100

C 9.78476600 10.33384700 13.82284400

C 8.95072300 10.59730500 12.70451800

C 7.06220100 10.42672600 13.97516700

H 5.97686900 10.46142400 14.01846000

C 7.79579500 10.16566500 15.14717900

H 7.27718900 10.00447300 16.08342300

C 9.15820700 10.11919900 15.06710500

H 9.76816200 9.91803900 15.94079200

**Lowest triplet state optimized structure**

Pt 5.40541500 7.29374500 8.58061700

Cl 3.37006100 7.03230400 9.65560300

Cl 5.99114700 5.08042800 8.94773800

S 8.62879300 11.06347000 9.99042100

O 7.83320500 12.24428100 10.15552200

O 9.57536100 11.00163500 8.91161100

N 4.91730700 9.22992100 8.19449200

N 7.60894300 9.80814500 9.82431700

N 7.16053400 7.55442700 7.58520200

N 7.60078400 10.68266300 12.78503700

C 4.15326400 9.47562600 7.12208600

H 3.84060900 8.61066700 6.55372400

C 3.77892100 10.75259800 6.76280000

H 3.15955100 10.89889000 5.88828900

C 4.20558700 11.81489600 7.54175500

H 3.92776000 12.83151500 7.29257500

C 4.99217000 11.55361300 8.64839100

H 5.35564500 12.35114500 9.28221700

C 5.34115300 10.24892900 8.95928000

C 6.19818500 9.95349300 10.15618400

H 6.10334200 10.75785600 10.88090400

H 5.86345600 9.03071700 10.63295400

C 8.20131100 8.49424800 9.59888700

H 7.63225900 7.76089800 10.17314400

H 9.21915800 8.49253900 9.99282500

C 8.25754600 8.07861400 8.15538700

C 9.42828900 8.22070300 7.42810200

H 10.29404000 8.64864100 7.91429500

C 9.47231100 7.83208500 6.10215800

H 10.38404500 7.94052000 5.52803500

C 8.33042800 7.30184900 5.52683300

H 8.30821900 6.97941400 4.49471000

C 7.19664100 7.17603500 6.30042400

H 6.28271200 6.75613700 5.90363900

C 9.57008700 10.76509100 11.44865600

C 11.00361200 10.68025600 11.32140700

H 11.44430900 10.78798500 10.34117100

C 11.75753900 10.47168900 12.42516000

H 12.83549300 10.40770600 12.34317700

C 11.14427700 10.32995900 13.73407900

H 11.76662400 10.16712200 14.60454400

C 9.73922800 10.39667400 13.88328200

C 8.92080800 10.61666000 12.73233900

C 6.98055600 10.53659600 13.99197600

H 5.89879900 10.59623400 13.97432900

C 7.66997800 10.32905800 15.15191600

H 7.14358600 10.22044700 16.09180800

C 9.08357900 10.25574900 15.10741500

H 9.65647000 10.08962900 16.01158000

SwissADME predictions of pharmacokinetic properties of L1, L2, C1 and C2.


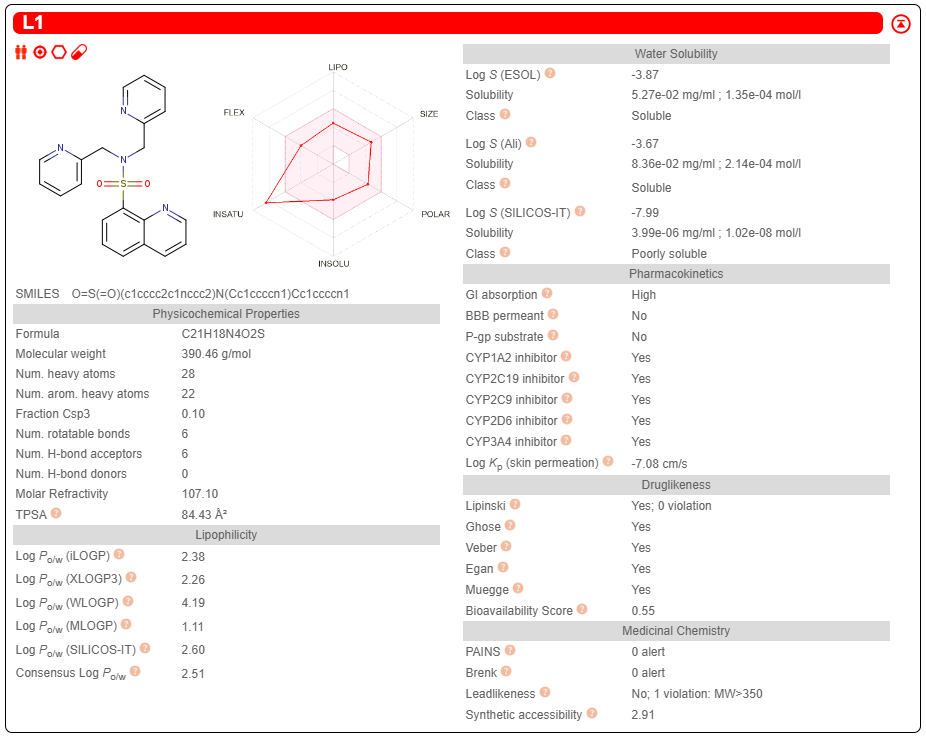


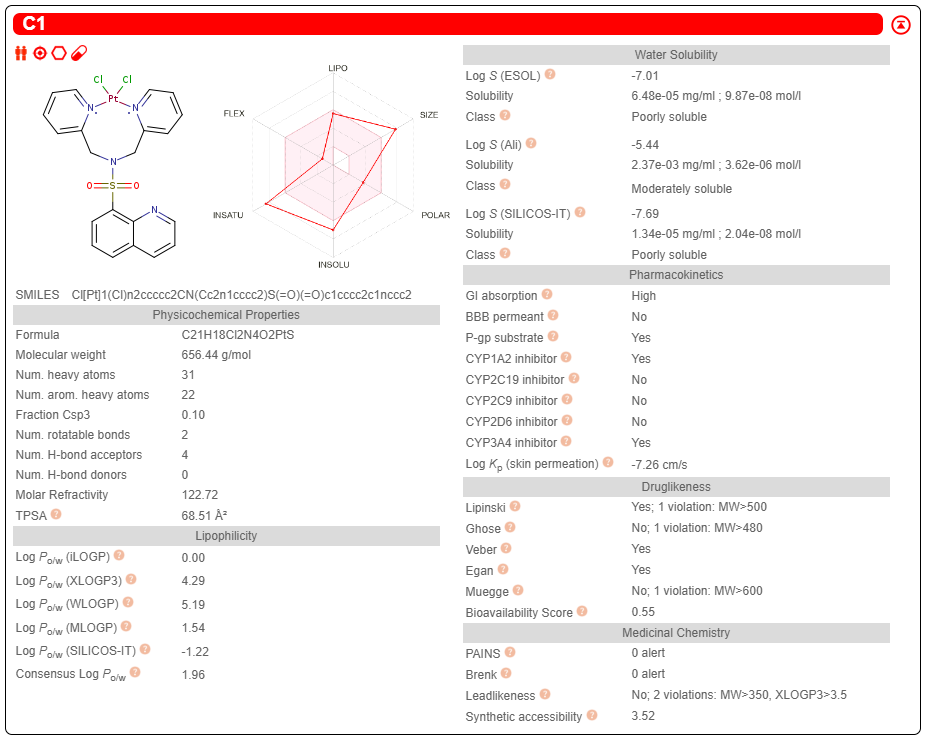


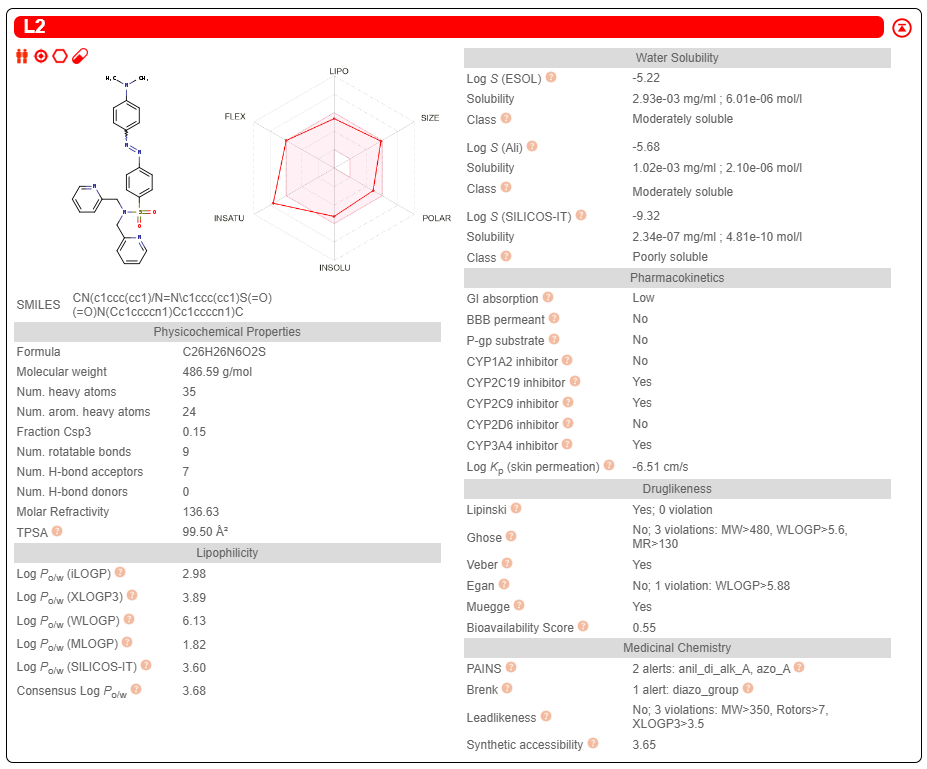


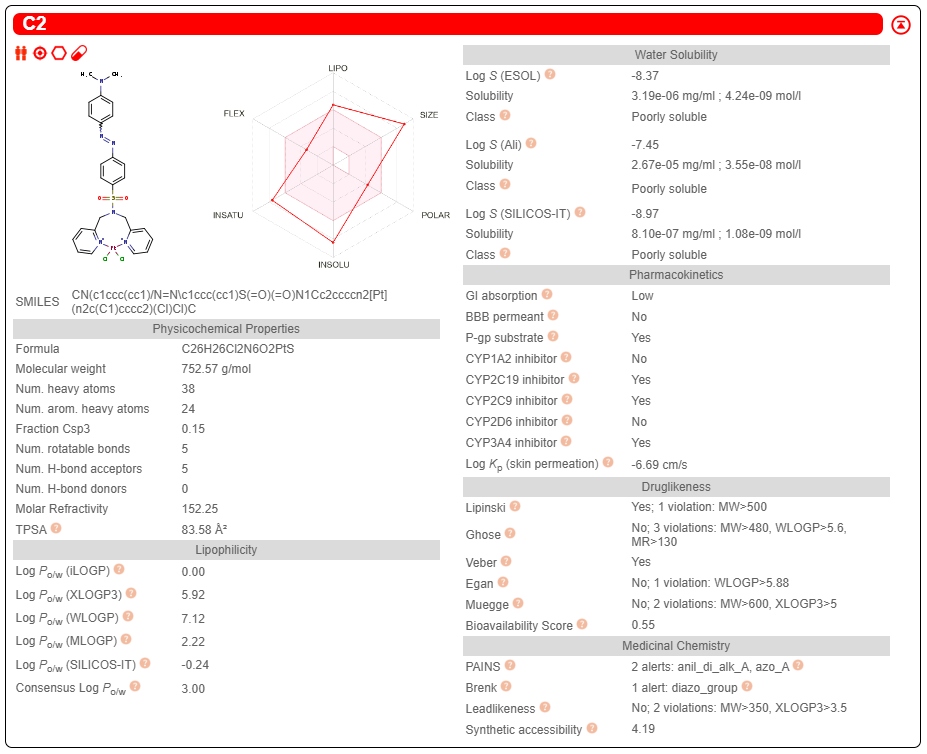


Figure S2: SwissADME predictions of pharmacokinetic properties of L1, L2, C1 and C2.

**Predicted Targets of L1, L2, C1, and C2. Highest probablility targets are mentioned here with their probablility scores.**


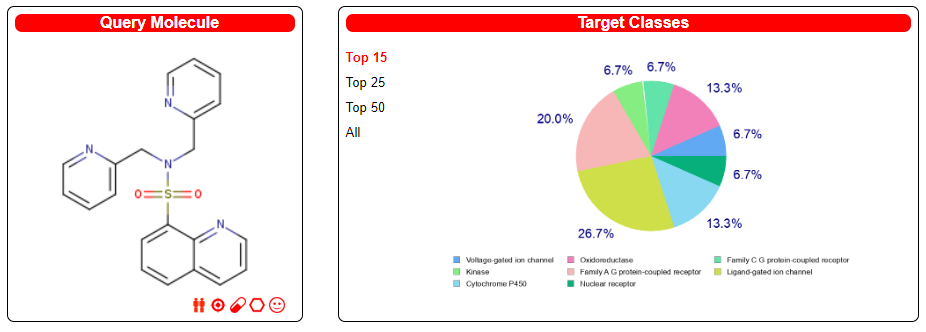

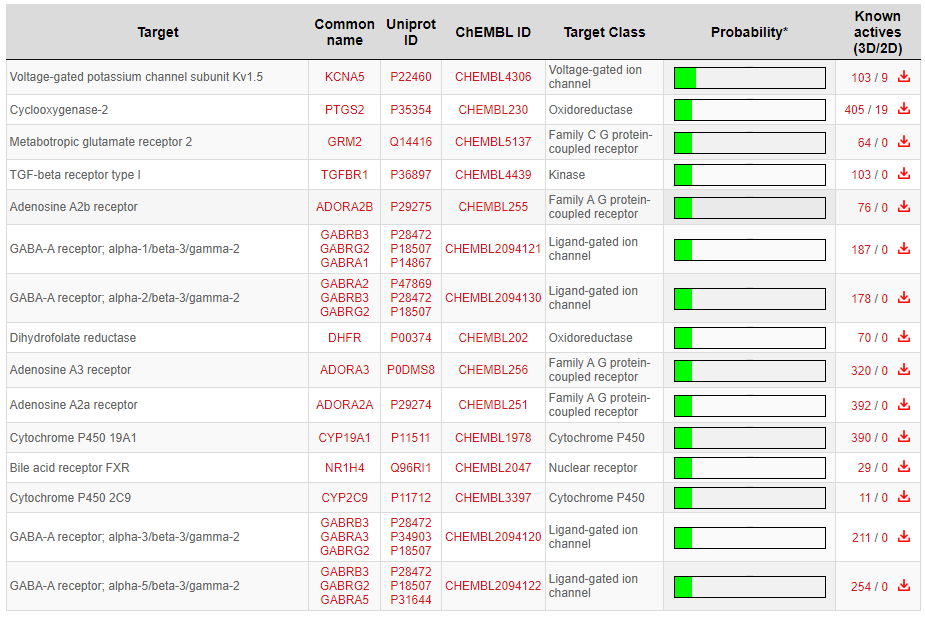


Figure S3: Predicted Targets of L1. Highest probablility targets are mentioned here with their probablility scores.


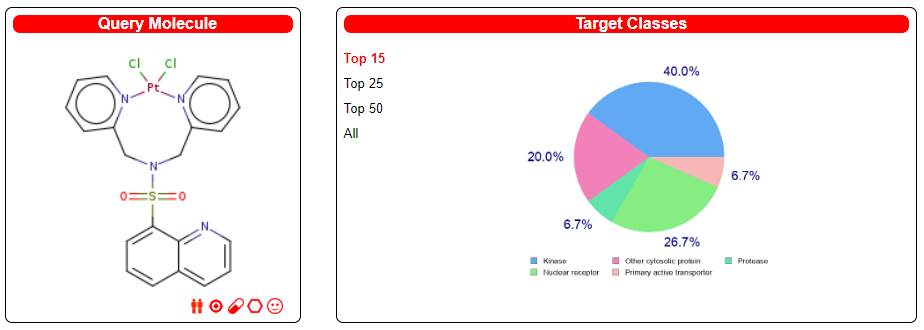

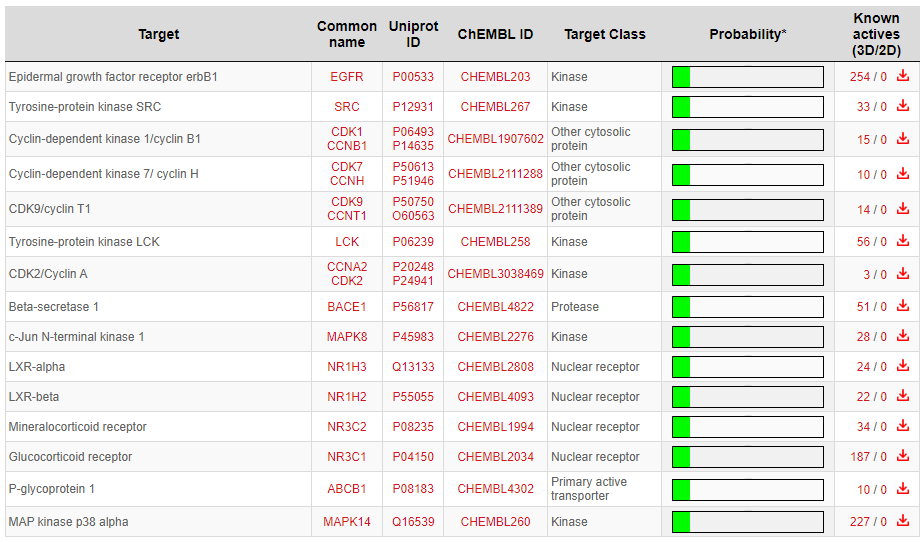


Figure S4: Predicted Targets of C1. Highest probablility targets are mentioned here with their probablility scores.


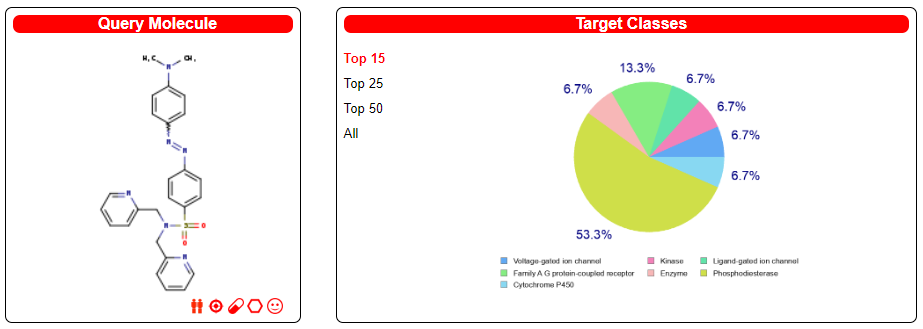

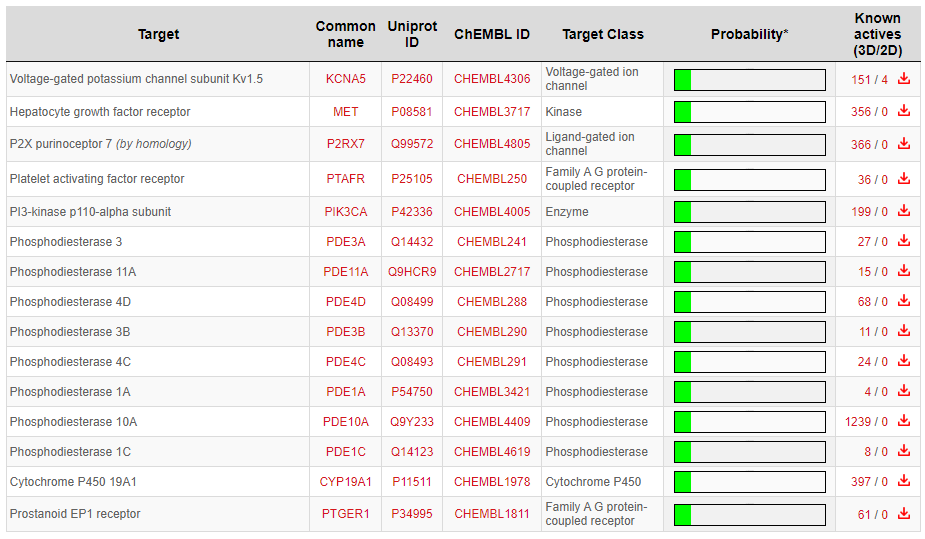


Figure S5: Predicted Targets of L2. Highest probablility targets are mentioned here with their probablility scores.


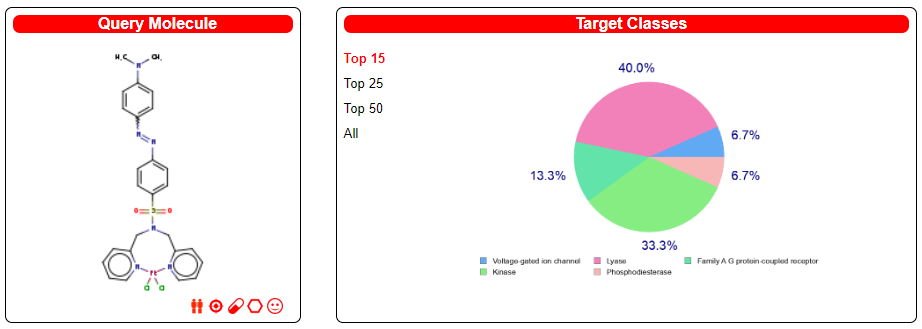

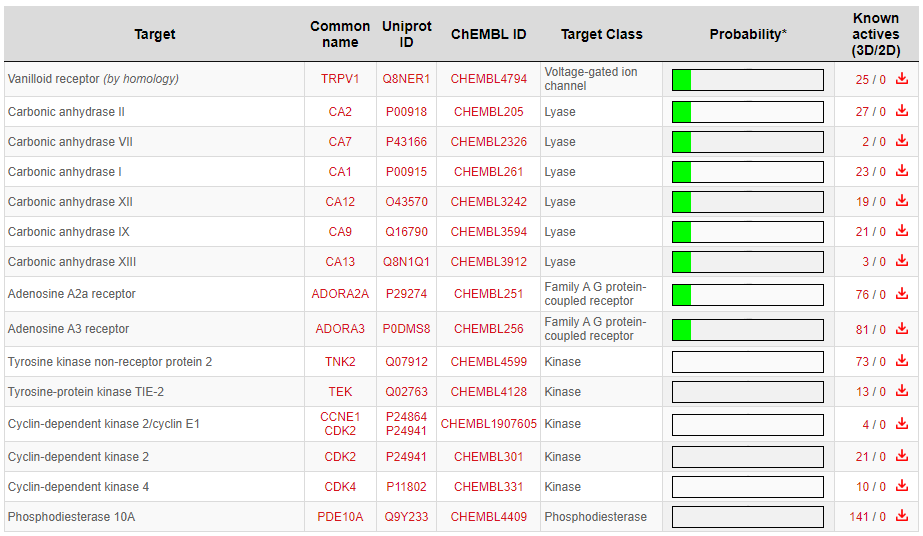


Figure S6: Predicted Targets of C2. Highest probablility targets are mentioned here with their probablility scores.


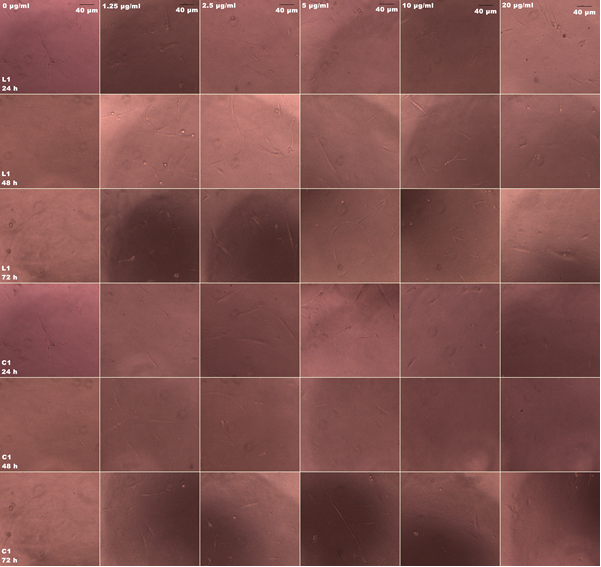


**Figure S7**: Morphology of MRC-5 cells after 24, 48 and 72hr incubating with L1 and C1 in increasing concentrations.


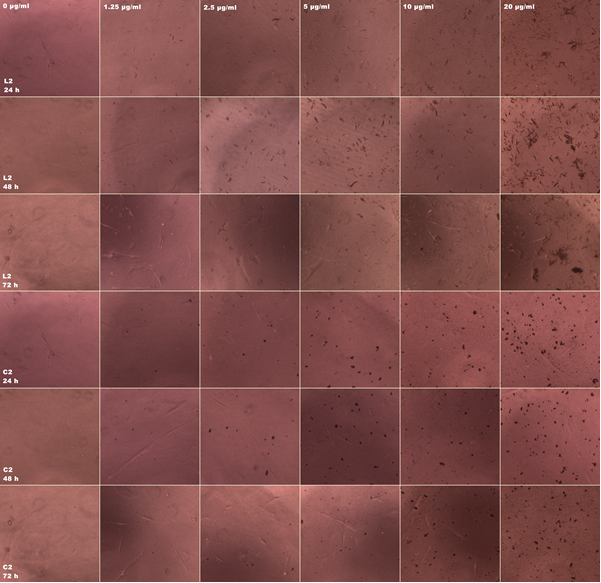


**Figure S8**: Morphology of MRC-5 cells after 24, 48 and 72hr incubating with L2 and C2 in increasing concentrations.


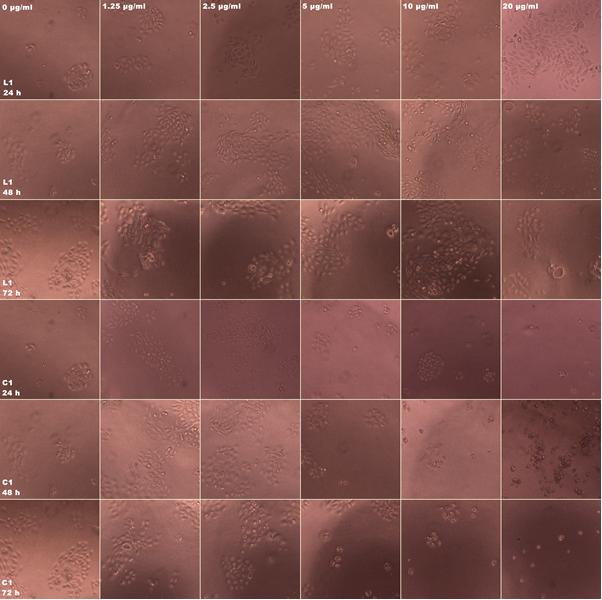


**Figure S9**: Morphology of human lung cancer cells (NCl-H292) after 24, 48 and 72hr incubating with L1 and C1 in increasing concentrations.


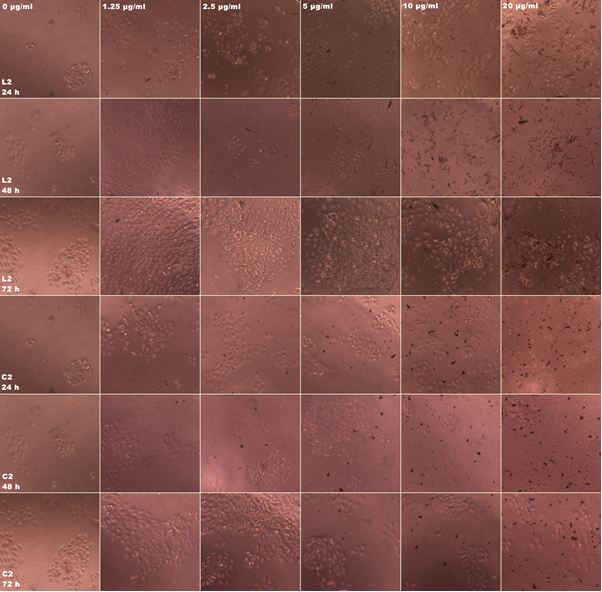


**Figure S10**: Morphology of human lung cancer cells (NCl-H292) after 24, 48 and 72hr incubating with L2 and C2 in increasing concentrations.
